# LncSL: A Novel Stacked Ensemble Computing Tool for Subcellular Localization of lncRNA by Amino Acid-Enhanced Features and Two-Stage Automated Selection Strategy

**DOI:** 10.3390/ijms252413734

**Published:** 2024-12-23

**Authors:** Lun Zhu, Hong Chen, Sen Yang

**Affiliations:** School of Computer Science and Artificial Intelligence Aliyun School of Big Data School of Software, Changzhou University, Changzhou 213164, China; zl@cczu.edu.cn (L.Z.); s22150812003@smail.cczu.edu.cn (H.C.)

**Keywords:** long non-coding RNA, stacking strategy, feature fusion, sequence analysis

## Abstract

Long non-coding RNA (lncRNA) is a non-coding RNA longer than 200 nucleotides, crucial for functions like cell cycle regulation and gene transcription. Accurate localization prediction from sequence information is vital for understanding lncRNA’s biological roles. Computational methods offer an effective alternative to traditional experimental methods for annotating lncRNA subcellular positions. Existing machine learning-based methods are limited and often overlook regions with coding potential that affect the function of lncRNA. Therefore, we propose a new model called LncSL. For feature encoding, both lncRNA sequences and amino acid sequences from open reading frames (ORFs) are employed. And we selected the most suitable features by CatBoost and integrated them into a new feature set. Additionally, a voting process with seven feature selection algorithms identified the higher contributive features for training our final stacked model. Additionally, an automatic model selection strategy is constructed to find a better performance meta-model for assembling LncSL. This study specifically focuses on predicting the subcellular localization of lncRNA in the nucleus and cytoplasm. On two benchmark datasets called S1 and S2 datasets, LncSL outperformed existing methods by 6.3% to 12.3% in the Matthew’s correlation coefficient on a balanced test dataset. On an unbalanced independent test dataset sourced from S1, LncSL improved by 4.7% to 18.6% in the Matthew’s correlation coefficient, which further demonstrates that LncSL is superior to other compared methods. In all, this study presents an effective method for predicting lncRNA subcellular localization through enhancing sequence information, which is always overlooked by traditional methods, and addressing contributive meta-model selection problems, which can offer new insights for other bioinformatics problems.

## 1. Introduction

Long non-coding RNAs (lncRNAs) are defined as RNA molecules exceeding 200 nucleotides in length and lacking the capacity for protein-coding [1]. LncRNAs have emerged as crucial regulators in various cellular processes [2], including the cell cycle [3], differentiation [4], metabolism [5], and disease mechanisms [6]. Unlike the initial belief that RNA mainly serves as an intermediary in transforming DNA into proteins, recent research underscores the multifaceted roles of RNA, particularly lncRNAs [7], in cellular functions. A recent study has found that, although a group of key long non-coding RNAs (lncRNAs) exhibit sequence and/or positional conservation in human embryonic stem cells, their processing mechanisms differ, leading to distinct subcellular localizations and functions in human cells [8]. The subcellular localization of lncRNAs is pivotal for their function, as it determines their molecular interactions [9] and regulatory capabilities [10]. For instance, lncRNAs in the nucleus may regulate gene transcription and chromatin modification [11], while those in the cytoplasm might influence mRNA stability and translation [12].

Wet laboratory methods such as in situ hybridization (ISH) and biochemical cell fractionation are commonly employed to determine the subcellular localization of lncRNA. ISH, which is one of the oldest and most widely used methods, and biochemical cell fractionation, which is considered more accurate, are notable examples [13]. However, the number of lncRNAs with a known localization remains insufficient given the vast number discovered. Experimental methods in wet laboratories are expensive, inefficient, and time-consuming. Consequently, computational methods are increasingly necessary to support and assist in this task. Several databases related to the subcellular localization of lncRNA have been established to promote the application of computational methods. These databases serve as benchmark sets for machine learning models. For instance, the RNALocate database records subcellular localization information for coding and non-coding RNA across 65 species [14]. Additionally, the lncATLAS database provides subcellular localization data for lncRNA from 15 different cell lines, measured in terms of relative concentration [15]. Among these cell lines, only the K562 cell line includes information on seven subcellular localizations of lncRNA, whereas other cell lines display only two locations, namely the cytoplasm and nucleus.

In molecular biology and genomics, open reading frames (ORFs) are pivotal segments extending from the start codon (typically ATG) to the termination codon in DNA or RNA sequences [16]. They serve as fundamental templates guiding protein biosynthesis, thus elucidating gene functions and potential protein types [17]. ORFs are crucial for cellular activities and are implicated in the regulation of diverse biochemical processes [18], encompassing cell cycle control, signal transduction, metabolic pathways, and cellular structure maintenance [19]. With today’s bioinformatics, which uses complex algorithms and big data analyses, ORF research goes beyond just looking at sequences. It can now find and predict possible ORFs in complicated genomes [20]. This not only aids in uncovering novel genes and functions but also in identifying disease-related genes and understanding disease mechanisms [21]. Exploring ORFs within lncRNAs presents a promising avenue. Although traditionally considered non-coding, recent studies suggest that certain lncRNAs may harbor unexplored ORFs potentially encoding functional short peptides or molecules regulating cellular processes [22]. This revelation offers fresh insights into lncRNA functionality and subcellular localization [23]. By scrutinizing ORFs, a comprehensive understanding of cellular molecular mechanisms is attainable, propelling advancements in disease treatment, drug development, and biotechnology [24]. The review by Xiao et al. highlights the potential of lncRNAs to encode functional micropeptides and their critical roles in disease mechanisms and cancer therapeutics. Additionally, their study provides a comprehensive list of tools for predicting and validating the coding potential of lncRNAs [25]. Hence, comprehending ORF roles in lncRNAs facilitates the prediction of lncRNA subcellular localization and functionality.

At present, some computational methods have been proposed to predict the subcellular localization of lncRNA. As far as we know, the first such predictor in the past was LncLocator [26]. LncLocator used an unsupervised deep learning model to create K-mer features and high-level abstract features. These features were then used to build four classifiers using support vector machines (SVMs) and random forests (RFs). Then, the four classifiers were combined using the stack integration strategy to obtain the final prediction results.

Gudenas et al. designed a deep learning model called DeepLncRNA [27]. The model uses K-mer features along with ENSEMBL transcript annotation and extra features that show the main lncRNA subtypes. A total of 1582 sequence-based features are obtained as input, and a feedforward multi-layer deep neural network architecture is used for training. Su et al. proposed ILoc-lncRNA, which converts lncRNA sequences into 8-mer frequency features and uses binomial distribution for feature selection [28]. ILoc-lncRNA combined 8-tuple nucleotide features into general PseKNC (pseudo-K-tuple nucleotide composition) through a binomial distribution method to predict the subcellular location of lncRNA, and LIBSVM 3.21 was used for prediction. Ahmad et al. developed Locate-R, used the l-mer composition and l-mer composition of n-gap as features, and selected the best 655 features to build the model. The model classifies four subcellular locations based on the local depth support vector machine [29].

**Figure 1:**
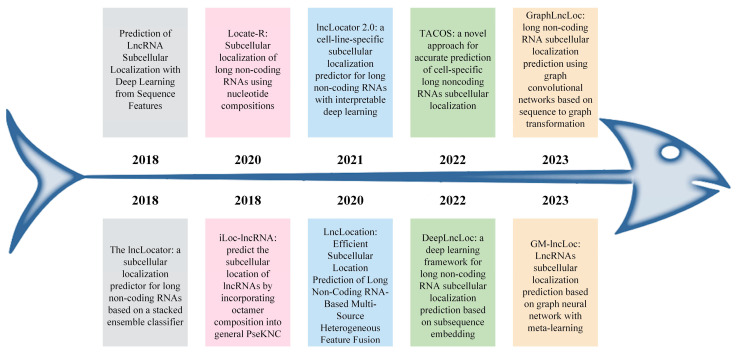
LncRNA subcellular location prediction technique.

Feng et al. developed an SVM-based model, LncLocation, and used the SVM model for training through the automatic encoder, the binomial distribution-based filtering method, and recursive feature elimination (RFE) to filter some features [30]. Lin et al. developed lncLocator 2.0 from the perspective of cell specificity, which uses natural language models to learn word embedding and then inputs it into convolutional neural networks, short-term memory, and multi-layer perceptron to classify subcellular localization [31]. In addition, Zeng et al. proposed DeepLncLoc, which is a deep learning framework based on a text convolutional neural network and uses subsequence embedding technology to code lncRNA sequences [32]. Jeon et al. proposed the tree-based stacking method, TACOS, comprehensively evaluated six tree-based classifiers with 10 different feature descriptors, and integrated the AdaBoost baseline model with the appropriate tree-based classifier for final prediction through the stacking method [33]. Cai et al. developed GM lncLoc, which used a graph neural network (GNN) to predict the subcellular location of lncRNA. This model extracts initial information from the lncRNA sequence, combines graph structure information to extract the features of lncRNA, and uses the training mode of meta-learning to obtain meta-parameters by training a series of tasks [34]. Li et al. proposed GraphLncLoc, a deep learning model based on a graph convolution network, to predict the subcellular localization of lncRNA [35]. GraphLncLoc changes lncRNA sequences into Bruijn graphs, which is different from previous studies that used K-mer frequency features to encode them. This means that graph classification is used instead of sequence classification. Figure 1 shows the development of techniques for predicting the subcellular location of lncRNA in recent years.

However, these methods still have limitations in the following aspects: (1) Small sample datasets are relied upon by Locale-R, DeepLncloc, and GraphLncLoc, with only a few hundred pieces of data, leading to overfitting and over-reliance on empirical parameters. (2) Shortcomings in feature engineering are present in existing models. Traditional nucleotide features are used by them all. Feature engineering is not present in some deep learning models, which rely on automatic feature extraction. A large amount of information about the sequence itself is ignored, leading to the loss of important information. (3) Existing predictors often rely on either a single model or the subjective selection of base learners when constructing stacked models. This approach typically involves choosing models based on prior experience or intuition, which may capture specific patterns in a particular dataset but struggles to generalize to new datasets due to a lack of systematic evaluation.

**Figure 2:**
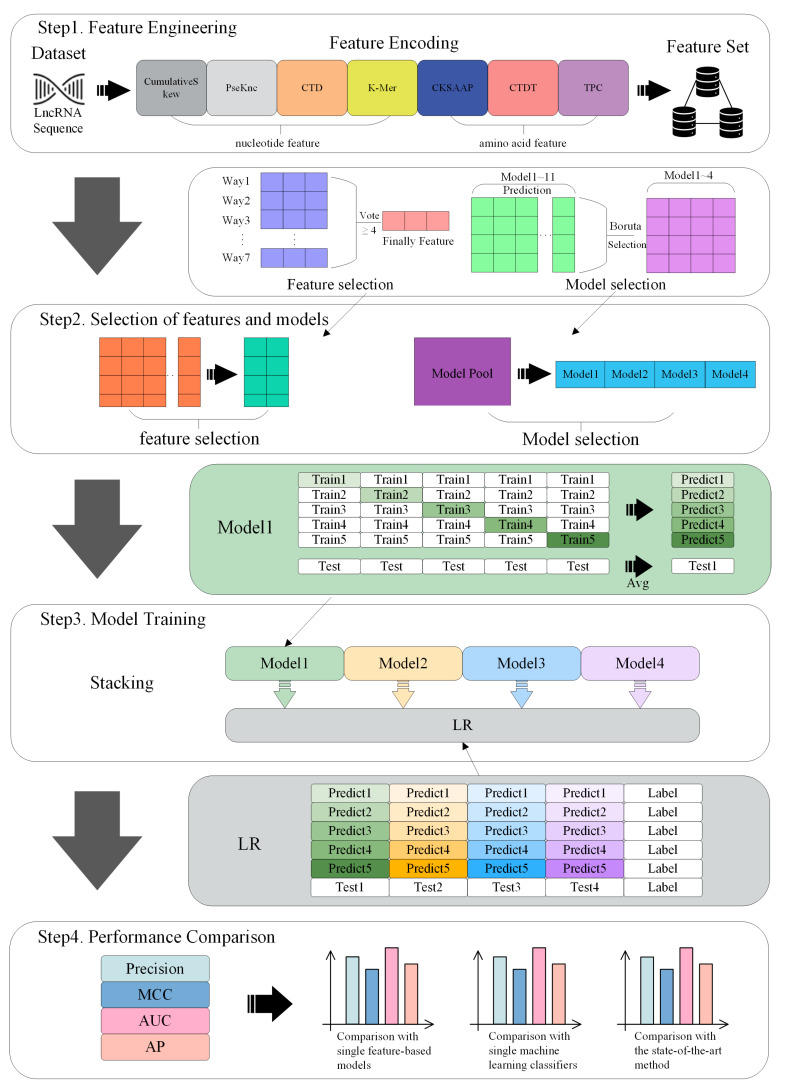
LncSL framework. Firstly, the dataset obtained is encoded by traditional nucleotide features and ORF-translated amino acid enhancement features; secondly, we conduct in-depth feature selection for the obtained high-dimensional feature matrix, select the features identified by more than four methods as our final feature space, train our feature space in the model pool, select the features through the output matrix composed of each output, and return the selected output to the model pool to find the four models that we finally use for stacking; thirdly, the four models selected by the model are used as the first basic classifier of the stack model, and the second meta classifier uses logical regression to form our LncSL model; Finally, LncSL is compared with the most advanced methods on several indicators.

The research framework of this paper is shown in Figure 2. Driven by these problems, a unique stacking strategy is adopted by LncSL. (1) A large number of datasets are used by LncSL, with thousands or even thousands of positive and negative samples, making the true distribution of data easier to capture and more robust and universally applicable laws easier to learn. (2) Almost all existing predictors have the same situation, that is, lncRNA regions that may contain coding potential are ignored. The combination of traditional nucleotide sequence features and the amino acid sequence features translated by ORFs is aimed at LncSL to better capture the high-level information ignored by traditional predictors. In addition, a variety of feature selection algorithms are used to ensure the most effective feature set is selected from the fused features. (3) Different from the previous single model or feature type method, an automatic model selection mode was developed using LncSL. A model pool containing multiple machine learning models was created, independent training for each model was conducted, and features for the training results were selected. The selected vector can return to the model pool to find the model suitable for the task, greatly reducing the intervention of human experience so that the model still performs well on a dataset that has not been seen before. In general, the performance of the model in various evaluation indicators, such as accuracy, F1-score, recall rate, etc., is not only improved using LncSL, but also, the interpretability and generalization ability of the model are enhanced.

The goal of this study is to develop a computational method that can accurately predict the subcellular localization of lncRNA. A higher accuracy than existing methods in both balanced and unbalanced test datasets is demonstrated using LncSL, indicating obvious advantages in dealing with lncRNA subcellular localization prediction. A new perspective for the functional research of lncRNA is provided with LncSL, as well as a new methodological perspective for the field of bioinformatics. The main characteristics and significant contributions of the method include:

1. Automatic ensemble learning techniques are employed by the proposed new model, LncSL, to model lncRNA sequences, successfully improving the predictive performance and interpretability of the model.
2. Multiple feature encoding methods were combined, including enhanced features of traditional nucleotide encoding and amino acid encoding in ORF translation, capturing information overlooked by traditional methods.
3. A stacking method for automatic model selection was proposed using LncSL, which autonomously selects the most suitable model from the machine learning model pool, reduces manual intervention, and improves predictive performance.
4. A scheme combining multiple feature selection methods was adopted in LncSL, and voting was used to select features recognized by more than four methods as the final feature space, significantly reducing the dimensionality of the feature space.
5. The proposed framework was trained and tested on a dataset of different scales using LncSL, including lncRNAs located in the nucleus and cytoplasm. Ablation experiments were also conducted to understand the contributions of ensemble algorithms, feature selection, and fusion feature encoding methods in the model.

## 2. Results

### 2.1. Comparison of Feature Extraction Methods

Choosing the appropriate feature extraction technology is crucial for building the most accurate prediction model to distinguish the subcellular location of lncRNA. To emphasize the benefits of integrating multiple features, this study has fused eight different feature coding methods. The selection of these coding methods includes the features based on the nucleotide sequence and the features derived from the amino acid sequence translated from the open reading frame in the nucleotide sequence. We fused the numerical vectors corresponding to the nucleotide feature encoding methods of each dataset to form a feature space called “All”. We fused the nucleotide feature encoding of each dataset with the numerical vectors corresponding to the derived amino acid-enhanced feature encoding method, forming a feature space called “All-Extended”.

#### 2.1.1. Comparison of Nucleotide Feature Extraction Methods

**Figure 3:**
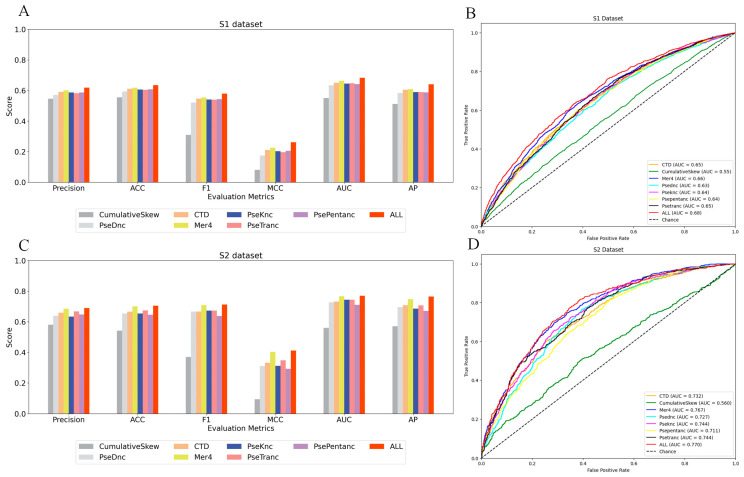
ROC curve and histogram of different nucleotide feature codes. (**A**,**C**) show performance bars for the different nucleotide feature encodings, and (**B**,**D**) are their ROC curves.

For feature selection, we employed CatBoost as the classifier and incorporated a validation dataset to implement an early stop mechanism. This allowed us to automatically optimize the number of iterations and achieve optimal performance. We then assessed the influence of various coding methods for single nucleotide features, as well as their combinations, on the prediction accuracy of lncRNA subcellular localization. The performance differences of various single and fusion feature coding schemes are revealed by the study of the prediction results of the two datasets, as shown in Table 1. Bold font for best effect. Furthermore, to ascertain the robustness of the prediction model across various feature extraction strategies, we employed ROC curves and histograms for assessment. Figure 3 displays the ROC curves and histograms of seven distinct approaches for extracting single nucleotide features and fusion features. These methods are evaluated on the training dataset, demonstrating their strong performance.

The information shown in Table 1 illustrates the variations among several feature extraction approaches in terms of several evaluation indicators, such as recall, precision, specificity, accuracy, F1-score, Matthew’s correlation coefficient, area under the curve, and average accuracy. Mer4 achieved the highest values in recall, precision, ACC, F1-score, MCC, AUC, and AP when using a single feature extraction method on dataset S1. The specific values were 51.5%, 60.1%, 61.8%, 55.5%, 22.6%, 66.4%, and 61.0%, accordingly. CumulativeSkew surpassed all others in the field of SP, achieving the highest figure of 84.6%. Furthermore, CTD and PsePentanc had comparable values to Mer4 across multiple indices. In the S2 dataset, the single feature extraction approach showed that Mer4 achieved the greatest values in recall, precision, ACC, F1-score, MCC, AUC, and AP. Specifically, Mer4 achieved a recall of 73.5%, accuracy of 68.5%, ACC of 70.1%, F1-score of 70.9%, MCC of 40.3%, AUC of 76.8%, and AP of 74.8%. CumulativeSkew exhibited the most outstanding performance in the SP indicators, attaining the highest value of 80.8%. The results demonstrate that, when used as a standalone feature extraction approach, Mer4 exhibits strong performance across many assessment factors.

However, the comprehensive feature extraction method outperforms the single feature extraction method in terms of prediction accuracy, as evidenced by evaluation indicators such as recall, precision, specificity, ACC, F1-score, Matthew’s correlation coefficient (MCC), area under the curve (AUC), and average accuracy (AP). In the S1 dataset, all the comprehensive features reached their highest values in all the indicators mentioned above, except for the CumulativeSkew feature. Specifically, the recall rate was 54.6%, precision was 61.9%, specificity was 71.2%, ACC was 63.5%, F1-score was 58.0%, MCC was 26.2%, AUC was 68.3%, and AP was 64.1%. When compared to the single feature extraction approach, the recall rate increased by 3.1% to 33%, precision increased by 1.8% to 7.3%, specificity increased by 0.5% to 2.2%, ACC increased by 1.7% to 7.9%, F1-score grew by 2.5% to 27%, MCC increased by 3.6% to 18.1%, AUC increased by 1.9% to 13.2%, and AP climbed by 3.1% to 12.9%. Comparable findings have been demonstrated in the S2 dataset. Except for SP indicators, all the above indicators have achieved their highest values in the comprehensive feature set. Specifically, the recall rate is 73.7%, precision is 69%, ACC is 70.5%, F1-score is 71.3%, MCC is 41.2%, AUC is 77%, and AP is 76.5%. When comparing the single feature extraction approach, the recall rate increased by 0.2% to 46.6%, the specificity increased by 0.5% to 10.9%, the ACC grew by 0.4% to 16.3%, the F1-score increased by 0.4% to 34.3%, MCC increased by 0.9% to 31.8%, AUC climbed by 0.3% to 21%, and AP increased by 1.7% to 19.4%. While the CumulativeSkew feature outperformed the comprehensive feature ALL in terms of specificity (SP), it was notably inferior to the ALL feature in other comprehensive assessment indicators, such as Matthew’s correlation coefficient (MCC) and average accuracy (AP). This disparity demonstrates that while several features may offer advantages in a singular measure when considering overall performance, the comprehensive attribute ALL is preferable due to its well-rounded and outstanding performance across multiple crucial evaluation indications. The primary benefit of ALL comprehensive features is derived from its integration of multiple feature information. By including data from several dimensions, it may capture the features of samples more fully, resulting in improved prediction performance across numerous assessment dimensions. Overall, the performance of all features is superior to that of single feature extraction approaches.

#### 2.1.2. Comparison of Fusion Amino Acid Feature Extraction Methods

**Figure 4:**
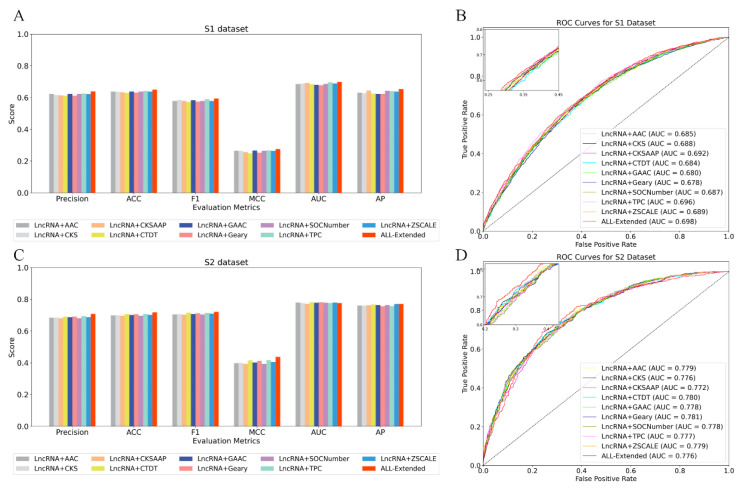
ROC curve and histogram of different fusion amino acid feature codes. (**A**,**C**) show the performance bars of different amino acid fusion feature codes, and (**B**,**D**) are their ROC curves.

Following a sequence of experimental investigations into the properties of nucleotides, we put forward a hypothesis, supported by existing research, suggesting that certain lncRNAs might possess sequences capable of encoding brief peptides. This finding posed a challenge to the conventional understanding of lncRNA as a non-coding element and indicated the need to incorporate these possible coding regions into prediction models. While these sequences alone are insufficient to constitute a whole protein, they can nevertheless impact the function and localization of lncRNA. Thus, we hypothesize that these prospective coding zones may encompass significant data overlooked by conventional techniques. We isolated these sections and converted them into amino acid sequences, allowing us to utilize a wide range of feature libraries that are based on amino acid sequences. Using this approach, we have found numerous specific attributes of amino acid sequences that are appropriate for our research. We integrated these features as derived attributes with the conventional nucleotide sequence feature to form a comprehensive feature space known as “All-Extended”. In our search for the most effective amino acid features, we utilized the CatBoost classifier and incorporated a validation dataset to implement an early stop mechanism. This allowed us to automatically optimize the number of iterations to achieve optimal performance. Additionally, we assessed the influence of different coding methods for single and combined amino acid features on the prediction of the subcellular localization of lncRNA. The performance disparities of different single and combination amino acid feature coding schemes are unveiled through the study of the prediction results from the two datasets (refer to Table 2). Bold font for best effect. Furthermore, to ascertain the robustness of the prediction model across various feature extraction strategies, we employed ROC curves and histograms for evaluation purposes. Figure 4 displays the receiver operating characteristic (ROC) curve and histogram of various individual and combined approaches for extracting amino acid features. These methods are fused together on the training dataset, effectively demonstrating their effectiveness.

Table 2 presents the variations in performance among various ways of extracting features generated from amino acids. The evaluation indicators used include recall, precision, specificity, ACC, F1-score, Matthew’s correlation coefficient, the area under the curve, and average accuracy. More precisely, among the data in set S1, the fused CKSAAP achieves the highest average accuracy index, reaching 64.4%. The fusion ZACALE feature achieved the maximum value in the specificity index, with a value of 71.8%. The integrated TPC features achieved optimal performance in terms of recall rate, precision, ACC, F1-score, Matthew’s correlation, and AUC, with values of 55.9%, 62.5%, 64.2%, 59.0%, 26.7%, and 69.6%, respectively. The fused CTDT features achieved the highest recall rate, F1-score, and AUC in dataset S2, with values of 74.1%, 71.5%, and 78.1%, respectively. The combined TPC features yielded the highest values in accuracy, specificity, ACC, and MCC, which were 69.4%, 68.0%, 70.8%, and 41.7%, respectively. The fused Zscale features achieved the greatest AP value, which was 77.0%. These results demonstrate that various feature extraction approaches exhibit variations in performance across diverse datasets.

This work investigated the combination of nucleotide and derived amino acid properties, broadening the analysis to include all derived amino acid traits. The optimal performance was attained through the fusion of three amino acid derivatives. The current nucleotide features form a strong foundation, and incorporating more amino acid features greatly expands the dimensions of the feature space, increases computational complexity, and introduces redundancy. To prevent these challenges, it is advisable to restrict the number of combined features. This approach ensures that the model remains versatile and effectively utilizes the available information. The three chosen derivatives, namely CKSAAP (local sequence patterns), CTDT (short-range sequence patterns), and TPC (chemical properties), enhance each other by capturing distinct aspects of lncRNA features, hence enhancing the accuracy of predictions. Therefore, combining these three qualities obtained from amino acids, based on nucleotide features, demonstrates the optimal performance.

The integration of our nucleotide features using the three amino acid-derived features CKSAAP, CTDT, and TPC yields the optimal outcome. The recall, precision, specificity, accuracy, F1-score, Matthew’s correlation coefficient (MCC), the area under the curve (AUC), and average precision (AP) for the S1 dataset are 55.5%, 63.9%, 73.2%, 65.0%, 59.4%, 27.6%, 69.8%, and 65.4%, respectively. When compared to other fusion approaches, either single or combined, the results show an increase of 0.9% to 2.7% on the Matthew’s correlation coefficient (MCC). Additionally, employing nucleotide features alone yields a 1.4% lower MCC compared to the fusion methods. The S2 dataset achieved the following percentages for recall, precision, specificity, accuracy, F1-score, Matthew’s correlation coefficient (MCC), the area under the curve (AUC), and average precision (AP): 73.5%, 70.8%, 70.1%, 71.8%, 72.1%, 43.7%, 77.6%, and 77.0%, correspondingly. In comparison to previous fusion procedures, the increase in MCC (Matthew’s correlation coefficient) ranged from 2% to 4.4%. Additionally, as compared to using nucleotide properties alone, there was a 2.5% increase in MCC. While the AUC value of our three amino acid fusion methods is marginally inferior to that of certain single amino acid fusion methods, other indicators outperform the single approach. Furthermore, upon closer examination of the ROC curve in the top left corner, it becomes evident that our three amino acid fusion methods outperform any individual method in accurately identifying the correct subcellular location of lncRNA. This demonstrates the model’s high accuracy in classification while minimizing error. Accurate classification is crucial in the subcellular localization analysis of lncRNA, as an incorrect localization might result in a flawed interpretation of its function. This, in turn, can have negative implications for future biological investigations and potential clinical applications. Hence, our approach of combining three amino acids is superior to that of combining a single amino acid.

These results highlight the importance of considering many feature types completely to achieve improved efficiency, rather than depending exclusively on a single feature encoding approach. The combination of nucleotide features offers a greater amount of nucleotide sequence information and yields a more effective prediction outcome, as compared to using an algorithm that encodes single nucleotide features. Furthermore, this study enhanced the prediction accuracy and applicability of the model by incorporating the amino acid sequence features derived from the translation of lncRNA regions that may possess coding potential, in addition to the conventional use of nucleotide features in traditional prediction methods. Overall, the method of feature fusion allows us to gather a more extensive range of sequence information, hence enhancing the precision of our predictions regarding the subcellular localization of lncRNA. Our technique demonstrates superior prediction ability compared to a single feature extraction algorithm, confirming its efficiency. This indicates that the amino acid sequence of ORF translation in lncRNA contains valuable information that is overlooked by previous methods.

### 2.2. Selection of Features and Models

In this work, we implemented a meticulous selection technique to carefully choose the feature space and the model utilized for stacking. Unlike traditional approaches for selecting features and models, we have implemented a novel automatic selection procedure that is not only more automated but also offers a more robust explanation. This method greatly enhances the model’s interpretability and generalization capability, hence improving its performance.

#### 2.2.1. Fusion Feature Selection

Conventional feature selection techniques depend on a solitary algorithm to reject features that have weak correlations, hence lowering the dimensions of the feature space and minimizing noise to enhance the performance of the model. Nevertheless, this strategy is constrained by the subjective choice of algorithms and the variability in their effectiveness. In order to address this issue, we employed seven commonly used feature selection algorithms, namely the variance threshold, F-test, recursive feature removal, tree model-based selection, L1 regularization, mutual information, and Boruta. Each algorithm autonomously chose features, leading to the creation of seven feature spaces. A voting procedure was employed to pick features that were present in at least four out of the seven techniques, which were subsequently included in the final feature space. This approach exploits the advantages of each method: the variance threshold effectively handles data with a large number of dimensions; the F-test identifies features that have statistical significance; recursive feature elimination takes into account the interactions between features; L1 regularization naturally selects features, which is particularly useful for high-dimensional data; mutual information captures nonlinear relationships; and Boruta automates feature selection without requiring extensive parameter tuning. The voting technique effectively combines these advantages to create a strong and comprehensive feature space.

The variance threshold method is a feature selection method based on feature variance. Its core idea is that if the variance of the feature is too small, it means that the feature has little change in the sample, so it may make little contribution to distinguishing different categories or forecasting results. Therefore, you can set a threshold to remove features whose variance is less than this threshold. The calculation formula of variance is shown in Formula (1):

(1) $$\sigma^{2}=\frac{1}{n}\sum_{i=1}^{n}{\left(x_{i}-\bar{x}\right)}^{2}$$

Among the variables, $x_{i}$ represents the feature value of the *i*-th sample, $\bar{x}$ is the mean of all sample values for this feature, and n is the total number of samples.

The F-test is based on the analysis of variance (ANOVA), which determines whether there is a significant difference in the mean between different groups by comparing the inter-group variance and intra-group variance. If the difference in mean between different groups is greater than the difference in mean within the group, we can assume that at least one group has a significant difference in mean compared to the other groups. The statistical calculation formula for F-test is shown in Formulas (2)–(4):

(2) $$F=\frac{MSB\left(Mean\;Square\;Between\right)}{MSW\left(Mean\;Square\;Within\right)}$$

(3) $$MSB=\frac{\sum_{i=1}^{k}n_{i}{\left({\bar{x}}_{i}-\bar{x}\right)}^{2}}{k-1}$$

(4) $$MSW=\frac{{\sum_{i=1}^{k}\sum_{j=1}^{n_{i}}\left(x_{ij}-{\bar{x}}_{i}\right)}^{2}}{N-k}$$

Here, $n_{i}$ is the number of samples in the *i*-th group, ${\bar{x}}_{i}$ is the mean of samples in the *i*-th group, $\bar{x}$ is the total mean of all samples, k is the number of groups, N is the total number of samples, and $x_{ij}$ is the *j*-th sample value in the *i*-th group.

The recursive feature elimination method is based on a base model that must provide an importance score for the features, such as the coefficients of a linear model or the feature importance of a decision tree model. Recursive feature elimination (RFE) relies on a base model providing feature importance scores, such as coefficients in a linear model or feature importance in a decision tree. In this case, a random forest is used. RFE recursively reduces the feature set size to identify the most important features. Initially, it trains the base model with all features, ranks them based on importance, removes the least important features, and repeats the process until the desired number of features is reached. This iterative process is formalized in Formula (5):

(5) $$F_{t+1}=F_{t}-\left\{j^{*}\right\},\mathrm{where}\;j^{*}=\arg\min_{j\in F_{t}}\omega_{j}\left(X_{F_{t}},y\right)$$

Here, $\omega_{j}\left(X_{F_{t}},y\right)$ represents the importance score of feature *j* trained based on the currently selected feature set $X_{F_{t}}$ and target variable y.

Model-based feature selection involves training a model and selecting features using the feature importance metrics provided by the model. This method can use any predictive model that can provide an estimate of feature importance, such as a random forest, gradient boosting machine, or any regularized linear model. We used a random forest with a threshold set to 1.5 × 10^−6^ and features larger than this value will be selected.

L1 regularization, also known as Lasso (Least Absolute Shrinkage and Selection Operator) regularization, is a widely used feature selection and regularization technique in statistics and machine learning. L1 regularization introduces an additional penalty term in the process of model optimization, which is the sum of the absolute values of all feature weights multiplied by the regularization parameter *λ*. For linear regression models, the loss function of L1 regularization can be expressed using Formula (6):

(6) $$L\left(w\right)=\frac{1}{2n}\sum_{i=1}^{n}{\left(y_{i}-w^{T}x_{i}\right)}^{2}+\lambda \sum_{j=1}^{m}\left|\omega_{j}\right|$$

Among the variables, n is the number of samples, w is the feature weight vector, $x_{i}$ is the feature vector of the *i*-th sample, $y_{i}$ is the target value of the *i*-th sample, m is the number of features, and λ is the non-negative hyperparameter that controls the intensity of regularization.

Mutual information (MI) measures the interdependence between two variables, indicating the amount of shared information. In feature selection, MI evaluates the correlation between features and target variables. Higher MI values signify more important features for predicting the target. MI is defined as the difference between the joint distribution of two random variables and their independent distributions. For two random variables X and Y, mutual information $I\left(X;Y\right)$ can be calculated using Formula (7):

(7) $$I\left(X;Y\right)=\sum_{x\in X,y\in Y}p\left(x,y\right)\log\frac{p\left(x,y\right)}{p\left(x\right)p\left(y\right)}$$

Among the variables, $p\left(x,y\right)$ is the joint probability distribution function of X and Y, while $p\left(x\right)$ and $p\left(y\right)$ are the marginal probability distribution functions of X and Y, respectively.

Boruta is a feature selection method based on random forests [36] that identifies meaningful features by comparing their importance with randomly generated shadow features. The core steps are as follows: (1) Shadow feature generation: shuffle dataset features to create shadow copies, disrupting their relationship with the target variable. (2) Train random forest: train a random forest model using both raw and shadow features, calculating feature importance. (3) Feature importance comparison: compare each original feature’s importance with the maximum importance of its shadow feature; features more important than their shadows are considered important. (4) Iteration process: repeat the steps, removing unimportant features after each iteration until all features are confirmed as important or unimportant. The process can be summarized as follows: let $\mathrm{Imp}\left(F_{i}\right)$ represent the importance of feature $F_{i}$ in a random forest, and $\mathrm{Imp}\left(S_{j}\right)$ represent the importance of shadow feature $S_{j}$. If feature $F_{i}$ satisfies Formula (8), it is considered important:

(8) $$\mathrm{Imp}\left(F_{i}\right)>\max\left(\mathrm{Imp}\left(S\right)\right)$$

where, if $\mathrm{Imp}\left(S\right)$ is the set of importance for all shadow features, then $F_{i}$ is considered important.

**Figure 5:**
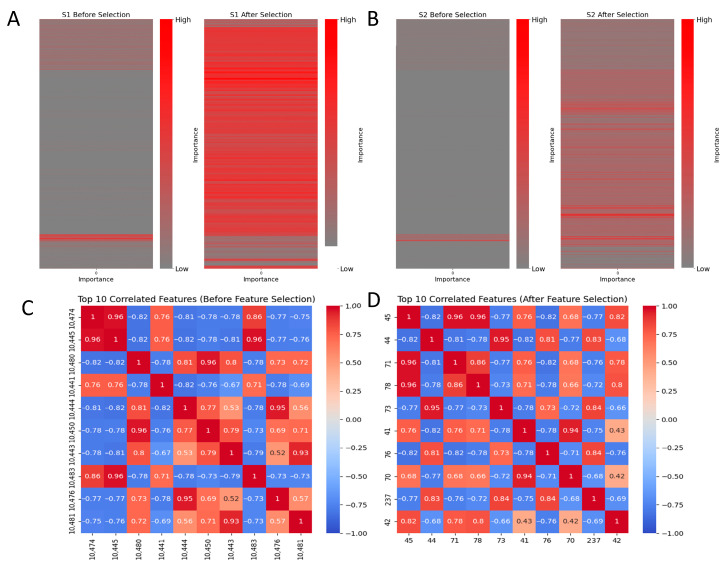
Comparison of importance and correlation heatmaps before and after feature selection. (**A**,**B**) show the feature importance heat maps after feature selection of different datasets, and (**C**,**D**) are their feature correlation heat maps. For the feature importance graph, the horizontal coordinate represents the feature importance, where the redder the color, the higher the importance, and the grayer the color, the lower the importance, and the vertical coordinate represents each feature. For the feature correlation matrix, the horizontal and vertical coordinates represent the number of ten features, and the darker the color, the higher the correlation.

Before conducting our comprehensive feature selection process, we combined features that resulted in a total of 12,099 dimensions. By employing this method, the number of dimensions in our S1 dataset was decreased to 258, while the number of dimensions in our S2 dataset was decreased to 385. We successfully decreased the number of dimensions in the feature space while still achieving high model accuracy. All indications remained positive, with even minor increments in MCC and ACC. Simultaneously, we significantly decreased the running speed while enhancing it by several orders of magnitude. The efficacy of our approach is demonstrated in Table 3. Bold font for best effect. We generated visual representations of the significance of heatmaps both before and after selecting features. Additionally, we examined the feature correlation heatmaps of the 10 most relevant features on average. These visualizations can be shown in Figure 5. Our multi-feature selection strategy has successfully eliminated the majority of low-importance features, resulting in a significant reduction in the dimension of the feature space. By utilizing the heatmap, we can observe that during the process of feature selection, the color distribution inside the heatmap becomes more evenly distributed, without surpassing the red or blue regions within the dataset. This suggests that there has been an enhancement in the independence across features and a decrease in the multicollinearity among features.

#### 2.2.2. Automatic Model Selection

After the feature space was established, we proceeded to construct models. Currently, the primary approaches for studying the subcellular localization of lncRNA can be categorized into two groups. The first group utilizes traditional machine learning techniques, including tree-based random forest and hyperplane-based support vector machine (SVM) techniques. The second group employs modern deep learning algorithms, such as convolutional neural networks (CNNs) and Long Short-Term Memory Networks (LSTMs). Nevertheless, conventional deep learning techniques have constraints when it comes to handling data with extended sequences. The length of our lncRNA sequence data might occasionally extend to tens of thousands or even hundreds of thousands, posing a challenge for conventional neural network architectures. Extended sequences can lead to a substantial rise in computational complexity, while ensuring efficient information transmission over long distances becomes a considerable hurdle. Conventional Recurrent Neural Networks (RNNs) and their modifications, such as LSTM and GRU, encounter the difficulty of capturing extensive dependence information, which restricts the model’s capacity to identify intricate patterns in lengthy sequences.

Due to the inadequacy of the deep learning architecture in our initial attempts, we opted for machine learning models. Machine learning offers superior interpretability compared to deep learning, as it gives an easier comprehension of anticipated dependent attributes and decision rules. In the realm of bioinformatics, specifically in the study of lncRNA, the ability to interpret models is of utmost significance as it aids in uncovering the relationship between sequence features and the subcellular localization of lncRNA. Once feature engineering has been adequately performed, conventional machine learning techniques can include these enhanced features in a more direct and adaptable manner.

While previous studies have successfully utilized a single machine learning model to predict the subcellular location of lncRNA and obtained favorable outcomes, it is important to note that relying solely on one model may overlook certain crucial information, leading to a decrease in prediction accuracy. In this study, we have implemented a stacking strategy for ensemble learning. While earlier research has utilized stacking approaches, the conventional stacking methods are too complex and subjective in selecting models. Typically, these methods only choose models that have superior predictive ability for stacking. While this approach may yield favorable outcomes, it diminishes the comprehensibility of the model. Consequently, we have devised an automated model selection technique that empowers machines to autonomously choose models.

We have created a model pool consisting of eleven frequently employed machine learning models. These models include logistic regression (LR), K-nearest neighbor (KNN), support vector machine (SVM), random forest (RF), Adaboost, gradient boosting (GB), extreme random trees (ExtraTrees), decision trees, XGBoost (XGB), LightGBM (LGBM), and CatBoost. Once the model pool building was finished, we used a grid search to identify the best parameters. We then proceeded to train each model in the model pool iteratively. Each model generates a unidimensional prediction outcome, which we then aggregate into a novel feature space. The Boruta feature selection algorithm was employed to conduct feature selection on the newly created feature matrix, to identify the most pertinent features (model outputs) for our purpose. This method has the capability to retrieve the relevant selected model from the model pool. Not only does this increase the understandability of stacked models, but it also greatly enhances their predictive accuracy. Upon implementing the aforementioned techniques on the S1 and S2 datasets, we made an intriguing observation: the S1 dataset yielded five selected models, namely random forest (RF), gradient boost (GB), lightweight gradient booster (LGBM), extreme gradient boost (XGB), and CatBoost. Conversely, the S2 dataset produced four selected models, specifically GB, LGBM, XGB, and CatBoost. It is important to mention that all models chosen in the S2 dataset were subsets of the models chosen in the S1 dataset, indicating a strong level of consistency in model selection across the two datasets. This outcome not only validated the efficacy of our approach but also underscored the fact that, despite the dissimilarity between the two datasets, the same model should be selected in theory as they address the same problem. Accordingly, we employed four models, namely GB, LGBM, XGB, and CatBoost, as the foundational models for the stacking model. The objective was to enhance the predicted accuracy and interpretability of the model by utilizing ensemble learning techniques.

#### 2.2.3. Integrate Features and Models After Selection

For our investigation, we chose four or more widely acknowledged traits from seven distinct feature selection approaches. The S1 dataset created a feature vector with a dimensionality of 258, while the S2 dataset created a feature vector with a dimensionality of 385. These feature vectors were used as the input feature space for our stacking model. Regarding model stacking, we utilized an automated model selection procedure to choose four fundamental models as the initial layer learners. These models include gradient boost (GB), extreme gradient boost (XGB), lightweight gradient boost (LGBM), and CatBoost. We utilized the StackingCVClassifier function from the mlxtend library, which is a set of machine learning extensions, to construct a stacking model. We employed these four algorithms as the foundational learners, with logistic regression serving as the subsequent layer meta-learner.

We selected logistic regression as a meta-learner because of its simplicity and interpretability. This allowed us to understand the final decision-making process of stacked models by examining the linear combination of explanatory weights (coefficients), even though the underlying base models were complex. We determined that the input features of the meta-model consisted of the predictions made by the base model, which typically had fewer features than the original dataset. This made logistic regression a suitable option for the meta-learners. When selecting a meta-model, logistic regression is frequently regarded as the most favorable option among stacked models because of its simplicity, efficiency, and strong probability estimation capability. However, the choice of meta-model ultimately depends on the particular job and dataset at hand.

During the stacking process, the projected probability output of the first layer basic model is utilized as the input feature for the meta-model. The training data for the meta-model are generated using five-fold cross-validation. Every individual base model is initially trained separately using the cross-validation training dataset and thereafter used to make predictions on the reserved validation dataset. The prediction outcomes, represented as probabilities, have been condensed into a new collection of features. These features are used to train logistic regression meta-models, which learn how to effectively combine the predictions of the basic model to enhance the overall predictive performance.

**Figure 6:**
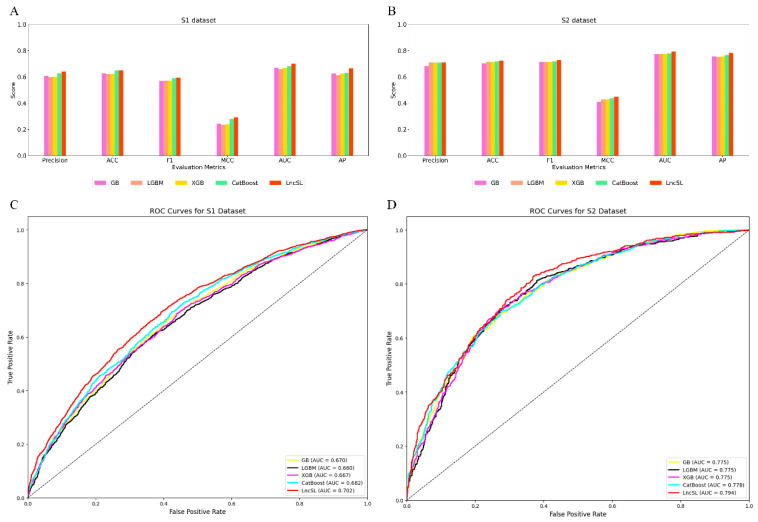
Histogram and ROC curve of different single models and LncSL. (**A**,**B**) show the performance comparison between different single models and LncSL, and (**C**,**D**) are their ROC curves.

The LncSL model was created by a meticulous process of feature selection and model combination. This approach not only enhances the model’s ability to make accurate predictions but also improves its ability to be understood and interpreted, surpassing the constraints of conventional stacking methods. Table 4 presents a comparison between LncSL and the single model. Bold font for best effect. We generated visual representations of the histogram and ROC curve, which can be seen in Figure 6.

### 2.3. Comparison with Existing Methods

To verify the performance of the LncSL predictor, we evaluated it using two datasets of different scales and compared it with several methods that have excelled in the past decade. Cao et al. (2018) [26] first predicted lncRNA subcellular localization using machine learning. Ahsan Ahmad et al. (2020) [29] introduced Locale-R, based on the Local Deep Support Vector Machine (LD-SVM) with l-mer and n-interval l-mer features. Yang Lin et al. (2021) [31] developed lncLocator 2.0, which uses end-to-end deep learning for cell-specific subcellular localization prediction. Zeng et al. (2022) [32] introduced DeepLncLoc, a deep learning framework using text convolutional neural networks and subsequence embedding techniques. Young Jun Jeon et al. (2022) [33] presented TACOS, a tree-based stacking method that combines multiple nucleotide features and integrates AdaBoost with a tree-based classifier. Min Li et al. (2023) [35] proposed GraphLncLoc, a model using graph convolutional networks to transform lncRNA sequences into Bruijn graphs, differing from K-mer frequency-based methods.

**Figure 7:**
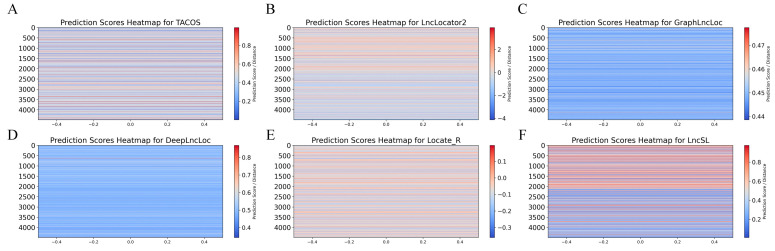
Heat map of prediction results of different models on the S1 dataset. (**A**–**F**) show the predicted heat maps of different models on the S1 dataset.

**Figure 8:**
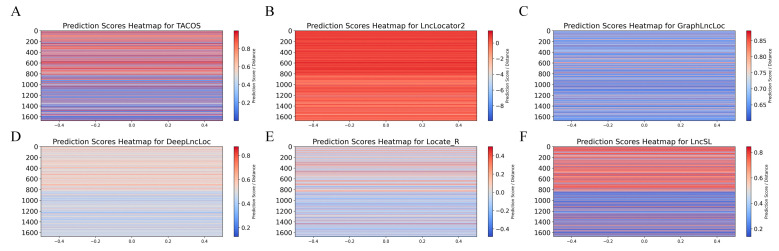
Heat map of prediction results of different models on the S2 dataset. (**A**–**F**) show the predicted heat maps of different models on the S2 dataset.

**Figure 9:**
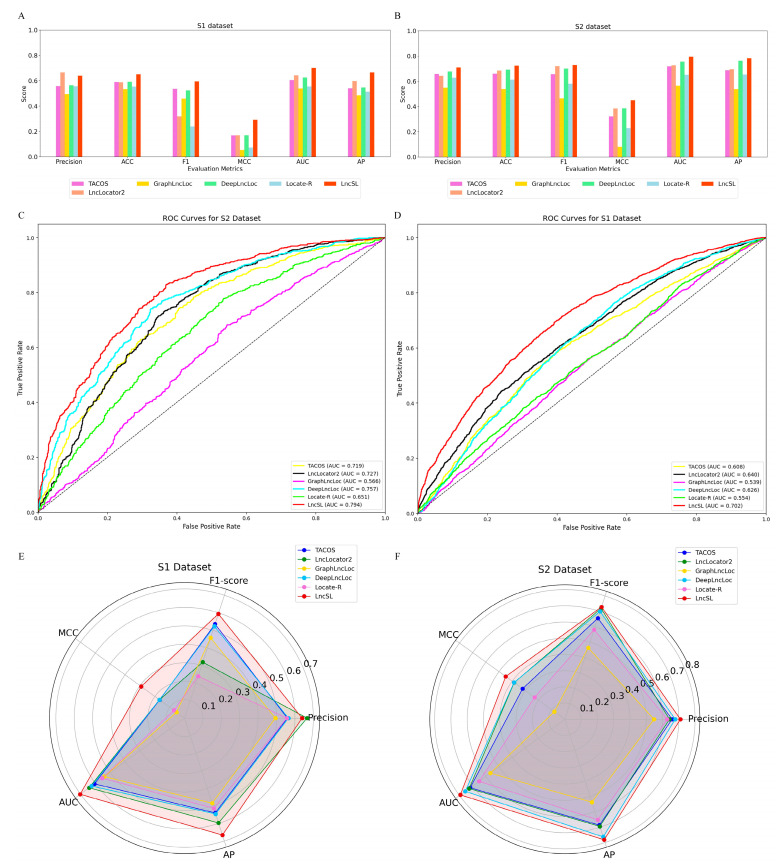
Bar charts, ROC curves, and radar plots of different models. (**A**,**B**) show a bar chart comparing the predictions of different models. (**C**,**D**) are their ROC curves. (**E**,**F**) are their radar maps.

Although existing methods have advanced in predicting lncRNA subcellular localization, they have limitations. Locale-R, DeepLncLoc, and GraphLncLoc rely on smaller datasets; LncLocator 2.0 has limitations in feature extraction; and TACOS is specific to certain cell lines. To address these limitations, we used two large-scale datasets (S1 with 22,439 data points and S2 with 8368 data points) and integrated multiple feature extraction methods for comprehensive sequence information. We included amino acid features from open reading frames (ORFs) as derivatives of nucleotide features to capture overlooked information. Additionally, we adopted automatic model selection to improve stability, accuracy, and interpretability. LncSL was evaluated on two datasets and compared with Locale-R, LncLocator 2.0, DeepLncLoc, TACOS, and GraphLncLoc, as shown in Table 5. Bold font for best effect. The performance analysis using histograms, radar plots, ROC curves, and heat maps (Figure 7, Figure 8 and Figure 9) indicates LncSL’s advantages over other methods.

### 2.4. Validation of Independent Test Dataset

**Figure 10:**
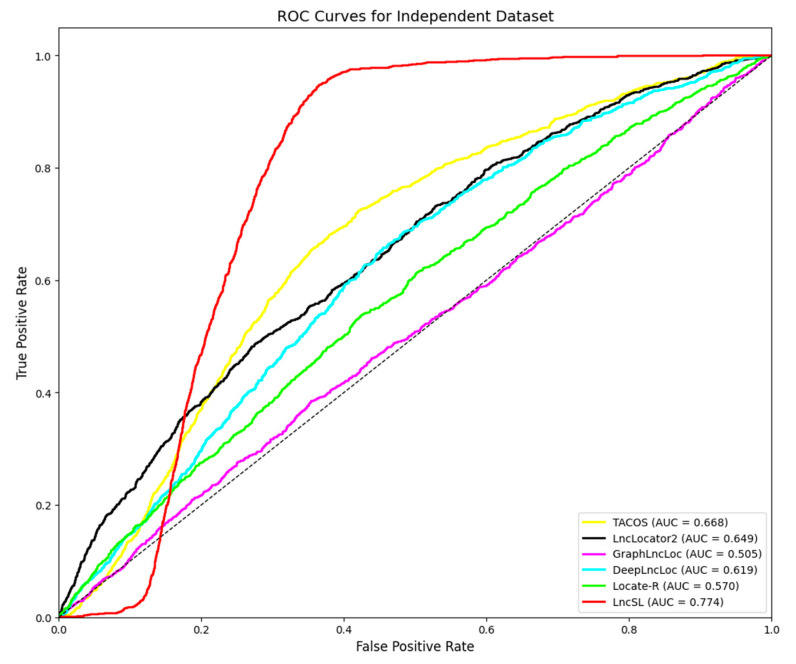
ROC curves of different predictors on independent test dataset.

In this study, we carefully selected 20% of the unused data from the S1 dataset as an independent test dataset to evaluate the performance of the lncRNA subcellular position predictor. This test dataset contains 1127 positive samples and 13,864 negative samples. To comprehensively compare its performance, we compared LncSL with five other prediction tools, including TACOS, LncLocator2, GraphLncLoc, DeepLncLoc, and Locale-R. The detailed results are listed in Table 6. Furthermore, we used ROC curves to evaluate the classification performance of each predictor. The ROC curves demonstrate the trade-off between sensitivity and specificity at different thresholds, as shown in Figure 10. This analysis aims to provide a comprehensive evaluation framework to accurately measure the performance of lncRNA subcellular localization predictors on the independent test dataset.

This study assessed five lncRNA localization prediction algorithms, namely TACOS, LncLocator2, GraphLncLoc, DeepLncLoc, and Locale-R, based on the data analysis shown in Table 6. Bold font for best effect. These methods exhibit varying performance across several evaluation measures, such as recall, precision, specificity, accuracy, F1-score, Matthew’s correlation coefficient, the area under the curve, and average precision. TACOS attained the greatest values of 22.1%, 15.3%, 66.8%, and 11.5% in F1-score, MCC, AUC, and AP, respectively. DeepLncLoc achieves the highest recall rate, reaching 59.4%. LncLocator2 demonstrated superior performance in terms of accuracy, specificity, and ACC, achieving values of 18.0%, 94.0%, and 88.1%, respectively.

The LncSL prediction model suggested in this study achieved the following results in all assessment indicators: 52.4%, 16.9%, 79.0%, 77.0%, 25.8%, 20%, 77.4%, and 14%. More precisely, when compared to current approaches, LncSL demonstrates notable enhancements. LncSL has demonstrated enhancements in recall, accuracy, specificity, ACC, F1-score, MCC, AUC, and AP by 0.4%, 2.9%, 5.0%, 4.6%, 3.7%, 4.7%, 10.6%, and 2.5%, respectively, when compared to TACOS. LncSL outperforms LncLocator2 in terms of recall, F1-score, MCC, AUC, and AP, with improvements of 36.1%, 8.7%, 9.2%, 12.5%, and 0.7%, respectively. Given the significant imbalance in the independent test dataset utilized in this study, it is worth noting that while LncLocator2 may have somewhat higher scores than LncSL in certain aspects, the MCC and F1-scores are more reliable indicators for evaluating the effectiveness of the model. LncSL has demonstrated a 9.2% enhancement in the MCC score and an 8.7% improvement in the F1-score when compared to LncLocator2. LncSL showed substantial enhancements in all assessment metrics compared to GraphLncLoc, with improvement rates of 26%, 8.8%, 3.1%, 4.9%, 13.4%, 18.6%, 26.9%, and 6.2%, respectively. LncSL outperformed DeepLncLoc in several metrics. Specifically, LncSL exhibited improvements of 6.3% in precision, 19.6% in specificity, 17.6% in accuracy, 7.8% in F1-score, 10.0% in MCC, 15.5% in AUC, and 3.9% in AP. In comparison to Locale-R, LncSL demonstrated enhancements of 36.3%, 6.0%, 12.8%, 15.4%, 20.4%, and 4.6% in recall, accuracy, F1-score, MCC, AUC, and AP, respectively.

In summary, the LncSL prediction model has demonstrated higher performance in many evaluation metrics when compared to existing approaches. The results highlight the possible use of LncSL in predicting the localization of lncRNA and its value as a robust technique.

Figure 10 shows that the LncSL predictor has a larger area under the ROC curve than other predictors that are available right now. The area under the ROC curve shows how well the model worked, and it shows that our LncSL prediction performed better than TACOS, LncLocator2, GraphLncLoc, DeepLncLoc, and Locale-R. The ROC graph shows that LncSL performs better than the other five methods by 10.6% to 26.9%. Overall, the LncSL predictor is thought to be the best way to guess where long non-coding RNA will be found within cells. The unevenness of the independent test dataset is what causes our model to have a longer starting lag than others in the curve. This difference makes it easy for the algorithm to find negative samples at lower limits. This means that fewer positive samples are correctly identified, and more positive samples are wrongly identified. Still, our model starts to perform better than others as the barrier goes up. This means that other models may have seemed to work better at first, but their higher position in the early part of the ROC curve may mean that their predictions are less accurate at lower levels. The larger area under the total ROC curve shows that our model performs better than all other forecast models.

## 3. Discussion

RNA sequencing technology has uncovered that the majority of transcripts in the human genome do not code for proteins, but instead are categorized as long non-coding RNA (lncRNA). These lncRNAs are often above 200 nucleotides in length. LncRNAs are increasingly recognized as having pivotal functions in various cellular processes, such as the cell cycle, differentiation, metabolism, illnesses, and viral infections. The subcellular location of long non-coding RNAs (lncRNAs) dictates their potential interactions with certain molecules, thereby influencing their biological roles. This paper presents a novel approach for predicting the subcellular localization of LncRNA. The method utilizes a stacking algorithm that combines nucleotide and amino acid information.

Two datasets, S1 and S2, were created by thoroughly analyzing the existing research literature, predictive models, and databases. These datasets varied in terms of their scales. The S1 dataset has 10,351 positive samples and 12,088 negative samples, whereas the S2 dataset consists of 4150 positive samples and 4218 negative samples. In order to guarantee the thoroughness and competitiveness of feature information, the CatBoost algorithm was employed to perform cross-validation on 14 different techniques of encoding features based on nucleotides. A number of approaches that demonstrated exceptional performance in capturing information content related to nucleotide sequences were chosen. Following a thorough analysis, five feature encoding approaches were chosen based on their strong performance in their respective domains or their high Matthew’s correlation coefficient (MCC) values. The mentioned factors include cumulative skewness, composition, transformation, and distribution (CTD), pseudo dinucleotide composition, pseudo-k-tuple nucleotide composition (PseKNC, k = 2, 3, 4, 5), and K-mer. The study revealed that employing a combination of cumulative skewness, CTD, PseKNC, and K-mer for feature extraction yields superior results compared to utilizing any single feature approach.

In addition, a novel technique for extracting features from amino acid sequences was proposed. This method is based on ORF translation and has been neglected by conventional approaches. This method efficiently catches the information that is overlooked by conventional methods by extracting open reading frames (ORFs) present in long non-coding RNA (lncRNA) sequences and translating them into sequences of amino acids. Experimental findings suggest that combining the five distinct feature encoding strategies outlined earlier, along with extracting amino acid features translated by ORFs as their derived enhancing features, can yield superior outcomes compared to employing a single feature. In addition, a meticulous selection of combined features was employed, and seven feature selection approaches were utilized to choose over four well-established features for the purpose of training. This greatly decreased the dimension of the feature space and enhanced the effectiveness of the model. The S1 dataset has a recall value of 55.6%, a precision value of 63.9%, an SP value of 73.2%, an ACC value of 65.0%, an F1-score value of 59.4%, an MCC value of 29.2%, an AUC value of 70.2%, and an AP value of 66.6%. The S2 dataset has a recall value of 74.9%, a precision value of 71%, an SP value of 70.0%, an ACC value of 72.4%, an F1-score value of 72.9%, an MCC value of 44.9%, an AUC value of 79.4%, and an AP value of 78.2%. This clearly illustrates the significance of multi-information fusion in enhancing the predictive capability of lncRNA in subcellular localization.

The creation of LncSL predictors employed a dual-layer prediction architecture, resulting in a substantial enhancement in prediction performance. This achievement is due to the effective integration of several feature extraction approaches and the benefits of the model. During the initial phase of LncSL, instead of using the conventional manual model selection approach, an automatic model selection strategy was employed. This process involved choosing four fundamental learners: XGBoost, LightGBM, CatBoost, and GBDT. These learners autonomously generate predictions and create a matrix for the main output, which is subsequently utilized as input for the secondary phase. The meta-model logistic regression method is used to combine the primary prediction results in the secondary stage of LncSL. The accuracy and reliability of the LncSL predictor were confirmed through comparative trials using conventional machine learning techniques (such as CatBoost, LightGBM, GB, XGBoost, and Adaboost) and existing predictors (TACOS, LncLocator2, GraphLncLoc, DeepLncLoc, and Locale-R). The increase in MCC values of LncSL on the S1 dataset varied from 1.3% to 23.8%, but on the S2 dataset, it ranged from 1.2% to 36.9%, demonstrating a considerable improvement compared to the comparison group.

The LncSL predictor was evaluated against five current predictors (TACOS, LncLocator2, GraphLncLoc, DeepLncLoc, and Locale-R) using an imbalanced independent testing dataset. The analysis revealed considerable enhancements in several important performance parameters. The LncSL predictor demonstrated enhancements ranging from 3.7% to 12.8% in F1-score, 9% to 23.1% in AUC, 4.7% to 18.6% in MCC, and an enhanced range of 0.7% to 6.2% in the AP value. Furthermore, the examination of receiver operating characteristic (ROC) curves and radar graphs amplifies the notable benefits of LncSL predictors in terms of prediction accuracy and dependability when compared to current predictors.

Finally, the dataset and code used in the study can be found on GitHub at https://github.com/ChenHongCCZU/LncSL (accessed on 20 December 2024). Regarding the dataset, the data on GitHub come from public databases and previous research and can be processed using the CD-HIT program as the basic data for research. For the code, the GitHub repository contains detailed information on feature methods, model architecture, hyperparameter settings, and other aspects used in this study, enabling other researchers to understand and replicate the research results. The GitHub link provides a specific URL to the GitHub repository, allowing other researchers to access the dataset and code used in this study to browse, download, reference, or utilize these resources.

## 4. Materials and Methods

### 4.1. Datasets

Previous studies have shown that strict datasets are essential for building reliable prediction models. To develop prediction models based on sequence information, lncRNA nucleotide sequence and location information are required. We have created two datasets, S1 and S2. The S1 dataset is mainly based on the work of Jeon et al [33]. in their TACOS research, which is available at https://balalab-skku.org/TACOS/download (accessed on 25 December 2023). The origin of this dataset is Lin’s research, lncLocator2. Lin et al. obtained variable-length nucleotide sequences from the GENCODE project [37] and location information from LncATLAS [38] to build a high-quality dataset. To determine the location of lncRNA, the cytoplasmic/nuclear relative concentration index (CNRCI) was used. In order to make it easier for classification, they filter out the data with CNRCI values in the range of [−1, 1] to facilitate classification. As a result, the preserved data, with a CNRCI value larger than 1 or smaller than −1, are significantly located in the cytoplasm or nucleus. Jeon et al. [33] modified the original data, combined these data with the training and validation dataset, and generated a new training dataset for each cell type. They adopted a CD-HIT [39] threshold of 0.8 to ensure the non-redundancy of the sequence. We combined these datasets to form a new training dataset and test dataset. Specifically, the combined training dataset contains 40,962 positive samples and 40,962 negative samples, while the test dataset contains 4650 positive samples and 40,670 negative samples. To avoid excessive data redundancy, we used the open-source CD-HIT program to remove the redundant processing of the data to ensure that the homology does not exceed 80%, so that the final S1 training dataset contains 10,351 positive samples and 12,088 negative samples. The source of dataset S2 is the research of Gudenas et al. [27], which is available from https://github.com/bgudenas/DeepLncRNA/ (accessed on 25 December 2023), and involves paired end chain specific RNA sequencing data from human cell lines as part of ENCODE project 14. The sample was cell-graded before RNA sequencing to separate the nucleus or cytosol. A total of 93 RNA-seq samples were obtained, of which 45 were from the cytosol and 48 from the nucleus. A total of 4380 cytoplasmic lncRNAs and 4298 nuclear lncRNAs were identified by applying the new log2-fold change threshold. The dataset is randomly divided into training, validation, and test datasets according to the proportion of 70%/15%/15%. We obtain the sequence from the Ensemble database according to the Ensemble ID provided by the author and use these IDs to obtain the location information as the identifier of the sequence to remove the data that cannot be retrieved. Finally, the S2 dataset contains 4150 positive samples and 4218 negative samples. The independent test dataset contains 1127 positive samples and 13,864 negative samples. Table 7 summarizes all datasets.

### 4.2. Methods

#### 4.2.1. Feature Extraction

In this study, we adopted a comprehensive feature extraction method, which not only includes the traditional nucleotide sequence features but also combines the amino acid sequence features translated from the ORF contained in lncRNAs. We first used the CatBoost algorithm [40] to select several features that performed well in our research tasks from the features based on nucleotide sequences, including cumulative skewness, composition, transformation, and distribution (CTD), pseudo dinucleotide composition (PseDNC), pseudo trinucleotide composition (PseKNC), pseudo tetranucleotide composition (PseTranC), pseudo pentanucleotide composition (PsePentaNC), and the frequency of K adjacent nucleotides (K-mer). For ORFs in lncRNAs, we use the orficy_core package [41] for extraction and then translate them into amino acid sequences through the Biopython package [42]. All possible ORFs between minlen and maxlen are found by entering a sequence of LncRNA through the start and stop codons. The two ORFs with the highest ratings are selected to score these possible ORFs, and if none are found, they are filled with zeros. In terms of the amino acid sequence, we used the ILearn package [43] to screen several features suitable for our task from a large number of amino acid features, such as CTDT (amino acid composition, transformation, and distribution features), CKSAAP (composition of k interval amino acid pairs), and TPC (tripeptide composition). By introducing the amino acid sequence features obtained from ORF translation, we effectively capture the parts that may be ignored by traditional nucleotide features.

In the context of the subcellular location prediction of lncRNA, utilizing amino acid sequence features offers several distinct advantages. Firstly, it enhances functional recognition by providing detailed insights into the structure and function of ORF-translated peptides, which are crucial for understanding the role of lncRNA within different cellular compartments. Secondly, amino acid sequences capture essential physicochemical properties such as hydrophobicity, charge, and molecular size, which are pivotal in determining lncRNA localization and function. Thirdly, these sequences facilitate the identification of intracellular signals and patterns essential for gene expression and regulation, thereby elucidating the roles of lncRNA in various cellular contexts. Fourthly, combining amino acid features with traditional nucleotide sequences significantly boosts the accuracy and robustness of lncRNA subcellular localization predictions. Lastly, this comprehensive approach enables a more thorough analysis of complex biological data, uncovering previously overlooked information.

In brief, by introducing amino acid sequence features, our method shows its advantages in theory and practice, providing a new and more accurate method for predicting the subcellular location of lncRNA. This is of great significance in the research and application of bioinformatics and opens a new horizon for the future research of lncRNA function and disease-related research.

##### Nucleotide Features

Cumulative skewness (CumulativeSkew)

Cumulative skewness is a statistical measure used to describe an uneven base distribution in DNA or RNA sequences, aiding in identifying features like replication start points and gene starting codons. In non-coding RNA research, especially lncRNA, it unveils unique structural traits, offering insights into function and subcellular location. This analysis primarily targets G-C and A-T deflections, yielding a list of two floating point numbers with a feature dimension of 2.

G-C deflection: Calculate the cumulative difference between *G* (guanine) and *C* (cytosine) bases. For each position *i* in the sequence, compute the difference between the number of *G* and *C* from the sequence start to that position, then divide by the total base number at that position. This calculation follows Formula (9):

(9) $$GC_{skew}\left(i\right)=\frac{\sum_{n=1}^{i}\left(count\left(G\right)-count\left(C\right)\right)}{i}\;$$

A-T deflection: Similarly, compute the cumulative difference between *A* (adenine) and *T* (thymine) bases. For each position *i* in the sequence, determine the difference between the number of *A* and *T* from the start of the sequence to that position, then divide by the total base number at that position. This calculation adheres to Formula (10):

(10) $$AT_{skew}\left(i\right)=\frac{\sum_{n=1}^{i}\left(count\left(A\right)-count\left(T\right)\right)}{i}\;$$

In lncRNA subcellular localization prediction, cumulative skewness unveils sequence-specific traits closely tied to its cellular distribution and function. Skewed patterns may signify localization within or outside the nucleus or involvement in regulatory processes. Integrating cumulative skewness with other bioinformatics features enhances sequence analysis for more precise lncRNA function and location prediction.

Composition/Transition/Distribution (CTD)

Composition, transformation, and distribution (CTD) features describe the distribution patterns of amino acids or nucleotides with specific structures or physicochemical properties in protein or nucleotide sequences [44]. They are crucial in bioinformatics for understanding lncRNA function and subcellular location. CTD features utilize seven physicochemical properties: hydrophobicity, normalized van der Waals volume, polarity, polarizability, charge, secondary structure, and solvent accessibility. The process involves (1) converting sequences into residue sequences with specific properties; (2) grouping the 20 amino acids into three groups for each physicochemical property based on Tomii and Kanehisa’s clustering [45]. CTD feature encoding generates feature vectors from three perspectives:

Composition: This feature describes the overall frequency of various amino acids or nucleotides in a sequence. For amino acid sequences, it calculates the frequency of 20 standard amino acids. For nucleotide sequences, it calculates the frequencies of the four bases: A, T, G, and C. The calculation formula is as follows:

(11) $$C_{x}=\frac{count\left(x\right)}{N}\;$$

Among them, *C_x_* represents the composition ratio of base (or amino acid) *x*, *count*(*x*) is the number of base (or amino acid) *x* in the sequence, and *N* is the total length of the sequence.

Transition: This feature measures the frequency of transitions between different types of amino acids or nucleotides in a sequence. In amino acid sequences, it includes transitions between polar and non-polar amino acids; in nucleotide sequences, it includes transitions from A to G or C to T. The calculation formula for AT transformation is as follows:

(12) $$T_{AT}=\frac{t_{AT}}{N-1}\;$$

Among the variables, *T_AT_* is the frequency of transition from A to T or from T to A, *t_AT_* is the number of times this transition occurs in the sequence, and *N* is the total length of the sequence. The transitions between other base pairs are similar to calculations.

Distribution: Distribution features focus on the spatial distribution of specific amino acids (or nucleotides) within a sequence. This typically involves calculating the frequency at which specific types of amino acids (or bases) first appear in the first 25%, 50%, 75%, and 100% positions of a sequence. The calculation formula is as follows:

(13) $$D_{x}\left(P\right)=\frac{position\left(x,p\right)}{N}$$

Among them, $D_{x}\left(P\right)$ represents the distribution frequency of the first appearance of base (or amino acid) *x* at the first *p*% position of the sequence, $position\left(x,p\right)$ is the specific position where base *x* first appears at that percentage position (corresponding to the length of the sequence), and *N* is the total length of the sequence.

The calculation method for CTD features varies based on the sequence type (protein or nucleotide) and target features. Compositional features tally amino acids or bases and standardize them into frequencies, resulting in four-dimensional features for nucleotides. Transformation features compute transitions between amino acids or nucleotides, yielding six-dimensional features for six nucleotide pairs. Distribution features identify positions where specific amino acids or bases first appear, resulting in 20-dimensional features by calculating five positions for each nucleotide. Thus, CTD features provide a 30-dimensional representation. In lncRNA subcellular localization prediction, CTD features reveal physical and chemical properties, aiding in inferring potential localization. Distinct composition and transformation patterns suggest interactions with specific organelles or structures, while distribution features illustrate spatial dynamics within cells. Combining CTD features with other bioinformatics data enhances prediction model accuracy and robustness.

Pseudo k-tupler Composition (PseKNC)

The encoded pseudo-k-tuple composition (PseKNC) contains the k-tuple nucleotide composition, which is used in this article as k = 2, 3, 4, 5 and can be defined in terms of Formulas (14) and (15):

(14) $$D={\left[d_{1},d_{2},\ldots ,d_{4^{k}},d_{4^{k}+1},\ldots ,d_{4^{k}+\lambda }\right]}^{T}$$

(15) $$\left\{\begin{array}{c} \frac{f_{u}}{\sum_{i=1}^{16}f_{i}+w\sum_{j=1}^{\lambda }\theta_{j}},\left(1\leq u\leq 4\right)\\ \frac{w\theta_{u-4^{k}}}{\sum_{i=1}^{4^{k}}f_{i}+w\sum_{j=1}^{\lambda }\theta_{j}},\left(4^{k}\leq u\leq 4^{k}+\lambda \right) \end{array}\right.$$

Among the variables, *λ* is the total count level (or hierarchy) along the correlation of nucleotide sequences, *f_u_* (*u* = 1, 2, …, 4*^k^*) is the standardized oligonucleotide frequency of $\sum_{i=1}^{4^{k}}f_{i}=1$, and *w* is the factor. *θ_j_* is defined in terms of Formula (16):

(16) $$\theta_{j}=\frac{1}{L-j-1}\overset{L-j-1}{\underset{i=1}{\sum }}\theta \left(R_{i}R_{i+1},R_{i+j}R_{i+j+1}\right),\left(j=1,2,\ldots ,\lambda ;\lambda <L\right)$$

The correlation function $\theta \left(R_{i}R_{i+1},R_{i+j}R_{i+j+1}\right)$ is defined in terms of Formula (17):

(17) $$\theta \left(R_{i}R_{i+1},R_{j}R_{i+j+1}\right)=\frac{1}{\mu }\overset{\mu }{\underset{v=1}{\sum }}{\left[P_{v}\left(R_{i}R_{i+1}\right)-P_{v}\left(R_{i+j}R_{i+j+1}\right)\right]}^{2}$$

Among the variables, *μ* is the quantity of physical and chemical indices, including six DNA indices (i.e., “up”, “roll”, “offset”, “slide”, “tilt”, “twist”) and six RNA indices (i.e., “up (RNA)”, “rotate (RNA)”, “offset (RNA)”, “slide (RNA)”, “tilt (RNA)”, “twist (RNA)”) which are set as the default indices for DNA and RNA sequences, respectively. $P_{v}\left(R_{i}R_{i+1}\right)$ is the value of the *v*-th (*v* = 1, 2, …, *μ*) physicochemical index of dinucleotide $R_{i}R_{i+1}$ at position *i*, and $P_{v}\left(R_{i+j}R_{i+j+1}\right)$ represents the corresponding value of dinucleotide $R_{i+j}R_{i+j+1}$ at position *j* + 1. The feature dimension is 4*^k^* + *λ*. We used four scenarios: *k* = 2, 3, 4, and 5, with *λ* all being 3. So, we obtained 19, 67, 259, and 1027 dimensional features.

K-mer

For K-mer descriptors, DNA or RNA sequences are represented as the frequency of occurrence of k-adjacent nucleic acids, which has been successfully applied in human gene regulatory sequence prediction [46] and enhancer identification [47]. The K-mer (k = 3) descriptor can be calculated using Formula (18):

(18) $$f\left(t\right)=\frac{N\left(t\right)}{N},t\in \left\{AAA,AAC,AAG,\ldots ,TTT\right\}$$

where *N*(*t*) is the number of K-mer type *t*, and *N* is the length of the nucleotide sequence. The feature dimension is 4*^k^*, and we take the value of *k* as 4, resulting in a 256-dimensional feature.

##### Amino Acid Features

CTDT

CTDT features offer a comprehensive sequence analysis by considering the physical and chemical properties of amino acids, such as hydrophobicity, polarity, and charge. This method extends traditional sequence analysis to include not only the basic composition but also the transformation and distribution of amino acids based on specific properties. The calculation of CTDT features involves the following. (1) Amino acid grouping: amino acids are divided into three groups based on different properties (Group1, Group2, Group3). (2) Conversion frequency calculation: for each adjacent pair of amino acids, conversion frequencies are calculated using Formulas (19)–(21), where Formula (19) calculates the conversion frequency from Group1 to Group2, Formula (20) from Group1 to Group3, and Formula (21) from Group2 to Group3:

(19) $$C_{1221p}=\frac{count\left(G_{1p}\to G_{2p}\right)+count\left(G_{2p}\to G_{1p}\right)}{N-1}$$

(20) $$C_{1331p}=\frac{count\left(G_{1p}\to G_{3p}\right)+count\left(G_{3p}\to G_{1p}\right)}{N-1}$$

(21) $$C_{2332p}=\frac{count\left(G_{2p}\to G_{3p}\right)+count\left(G_{3p}\to G_{2p}\right)}{N-1}$$

(3) Feature vector construction: for each physical and chemical attribute, generate a feature vector containing the three transition frequencies mentioned above.

We calculated three sets of ratios using 13 physical and chemical properties, including hydrophilic and hydrophobic indicators, van der Waals volume, polarity, polarizability, charge, secondary structure tendency, and solvent contact area, resulting in 39-dimensional features. CTDT features are crucial for lncRNA subcellular localization prediction, reflecting both the basic sequence composition and complex interactions of amino acid properties in ORF-translated peptide sequences. This detailed analysis aids in predicting lncRNA locations within cells, providing key insights into their function and biological roles.

CKSAAP

The CKSAAP method [48], widely used for encoding protein sequence features, excels in protein function prediction and classification tasks, showing robust performance in various bioinformatics applications. In predicting lncRNA subcellular localization, CKSAAP features provide crucial insights into the local structure and functional properties of sequences translated by ORFs. By analyzing the composition of amino acid pairs with specific intervals, CKSAAP reveals distinct patterns related to lncRNA localization and function. It calculates the frequency of all possible amino acid pairs with a predefined gap *k*. For *k* = 0, it computes 400 pairs (e.g., AA, AC, AD, YY...), as defined in Formula (22):

(22) $$\left(\frac{N_{AA}}{N_{total}},\frac{N_{AC}}{N_{total}},\frac{N_{AD}}{N_{total}},\ldots ,\frac{N_{YY}}{N_{total}}\right)400$$

Each descriptor value represents the composition of the corresponding residue pairs within the peptide sequence. To illustrate, when the residue pair AA appears m times in the peptide sequence, the composition of the AA residue pair is determined by dividing m by the total number of residue pairs with zero intervals within the peptide sequence (total *N*). We take *k* as 5 here, so we obtain a total of (500 + 1) × 400 = 2400 dimensional features.

Tripeptide Composition

The tripeptide composition (TPC) method analyzes amino acid sequence position and information [49], revealing biological signals relevant to subcellular localization. Certain tripeptide combinations can indicate specific cellular subregions, providing broader biological insights. Sequences are standardized to 300 amino acids, padded, or truncated as needed. Each amino acid is mapped to a unique index, and feature vectors of 8000 dimensions, representing all possible tripeptide combinations, are initialized. By traversing every three consecutive amino acids, the corresponding feature vector index for each tripeptide is calculated using the assigned index mapping, as shown in Formula (23):

(23) $$Index=i_{1}\times 20^{2}+i_{2}\times 20+i_{3}$$

Among them, $i_{1}$, $i_{2}$, and $i_{3}$ are indices of three consecutive amino acids. Subsequently, the value of the feature vector at that index is increased. Finally, the feature vectors are normalized to reflect the relative frequency of the tripeptides rather than their absolute quantity. The normalized feature vector calculation formula is shown in Formula (24):

(24) $$Normalized\;Feature=\frac{Feature\left[i\right]}{\sum_{j=1}^{8000}Feature\left[j\right]}$$

Among them, $Feature\left[i\right]$ is the non-normalized tripeptide frequency. The TPC method fully captures the local structural features of amino acid sequences by comprehensively considering all possible tripeptide combinations. A significant advantage of this method is that it does not rely on any prior knowledge of the protein’s known structure or function, making it highly suitable for the feature extraction of amino acid sequences from potential open reading frames (ORFs) in lncRNA translation.

#### 4.2.2. Proposed Methodology of LncSL

**Figure 11:**
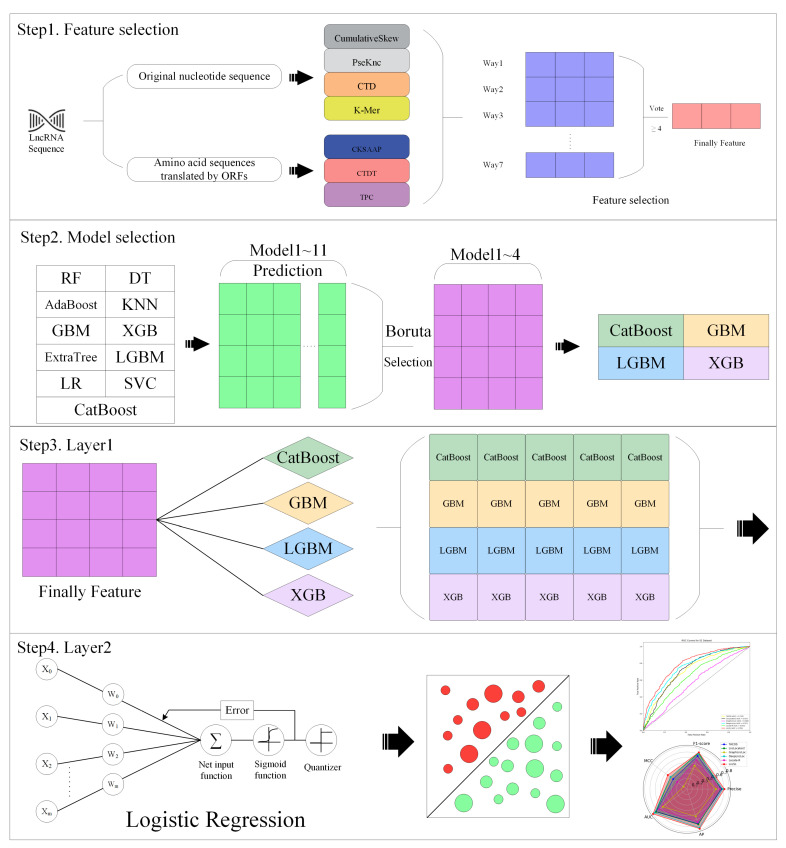
Automatic model selection stacked integrated learning LncSL. Initially, seven types of lncRNA sequences were isolated by utilizing conventional nucleotide features and ORF-translated amino acid-improved features. Subsequently, deep feature selection was performed on the obtained high-dimensional feature space. Furthermore, the feature space underwent sequential training using a pool of 11 models, resulting in the formation of an output matrix composed of these 11 output vectors. Feature selection was performed on the output matrix, and the resulting matrix was then returned to the model pool to identify the four most appropriate models for the task. Next, the input for the stack model was the final feature space, and the first layer of the stack model utilized five-fold cross-validation with the four models chosen by the automatic model. Ultimately, the outcomes obtained from the initial layer were forwarded to the meta-learner, logistic regression, to obtain the probability of prediction for the outcome.

Ensemble learning is a robust technique where numerous weak classifiers are amalgamated to create a more potent classifier. Bagging [50] and boosting [51] are the two usually employed comprehensive learning methods. In bagging, a random process is used to generate subsets of data, and weak classifiers are trained on each subset. Majority voting rules are then employed to amalgamate these weak classifiers. Conversely, boosting utilizes an iterative methodology to enhance prediction precision by adjusting the weight distribution of samples and training new weak classifiers using misclassified samples. Stacked ensemble learning employs numerous underlying models to make predictions. Initially, four fundamental models are trained, and their training outcomes are fed into a meta-model to produce the ultimate prediction. This article introduces a stacked ensemble learning LncSL predictor. The details of the specific model are shown in Figure 11. To make better predictions, diverse methods for encoding features and basic learners were combined, effectively utilizing the various features and information present in the sequence. A dual-layer prediction architecture was employed, harnessing the benefits of several methodologies and models to enhance the forecast accuracy.

In the first layer of LncSL, seven distinct feature encoding approaches were employed to capture fundamental traits and information within the sequence. Conventional methods for forecasting the subcellular localization of lncRNA solely rely on the lncRNA sequence, disregarding the possibility that certain portions of the lncRNA may possess coding capabilities. This investigation considered not only the lncRNA sequence but also the amino acid sequence features of the ORF translation found within the nucleotide sequence. The combination of nucleotide and produced amino acid sequence features was utilized to enhance prediction accuracy and improve the generalization ability. The methods for encoding nucleotide features included cumulative skewness, composition, transformation, and distribution (CTD), pseudo K-nucleotide composition (PseKNC), and the frequency of K adjacent nucleotides (K-mer). The encoding techniques for amino acid properties encompass CTDT (composition, transformation, and distribution features of amino acids), CKSAAP (composition of k-interval amino acid pairs), and TPC (tripeptide composition). The feature vectors obtained from various feature extraction algorithms were merged into a matrix, resulting in a high-dimensional feature matrix. Various feature selection strategies were employed to identify the most appropriate features for the model. Unlike conventional feature selection approaches, seven feature selection algorithms were used to choose the created feature matrix, resulting in seven feature selection outcomes. Features recognized by more than four techniques were included in the feature space, based on the index of the seven feature selection outcomes. The final feature matrix, which included these highly recognized traits, was then used for model training. Let *X* be the feature matrix formed by combining different encoding methods. *F_i_* represents the set of features selected by the i-th feature selection algorithm, where *i* = 1, 2, 3, …, 7. The final feature set *F* used for training is given by Formula (25), where δ is the indicator function:

(25) $$F=\{f|\sum_{i=1}^{7}\delta (f\in F_{i})>4\}$$

When it comes to choosing basic learners, a model pool consisting of eleven machine learning models was developed: AdaBoost, LR, ExtraTrees, DecisionTree, KNNeighbors, SVM, RF, GB, XGBoost, LightGBM, and CatBoost. Unlike classic stacking methods that are challenging to clarify, this approach is more straightforward. Separate training sessions were conducted for each of these models, with the output of each model considered as a distinct feature vector. These feature vectors were then combined to create a new feature matrix. Each model *Mj* (where j = 1, 2, …, 11) was individually trained using the chosen feature set *F*. The result of each model was treated as a distinct set of features, which were then combined to form a new set of features represented by the feature matrix *Z*. Subsequently, the matrix underwent additional feature selection to identify the most appropriate features for the meta-model. Let *hj*(*X*) be the output of model *Mj*. *Z* may be expressed using Formula (26):

(26) $$Z=[h_{1}(X),h_{2}(X),\ldots ,h_{11}(X)]$$

Afterward, feature selection algorithms were employed to choose the most appropriate features from the pool of models. By considering these features, the result associated with a specific model could be obtained, allowing for the selection of the model most suitable for the objective. Both the S1 and S2 datasets underwent identical operations. From the S1 dataset, five models (RF, GB, XGBoost, LightGBM, and CatBoost) were chosen. Additionally, from the S2 dataset, four models (GB, XGBoost, LightGBM, and CatBoost) were selected. Remarkably, all four models chosen from the S2 dataset were also included in the results of the S1 selection, conclusively demonstrating the efficacy of this approach. Since it is necessary for models chosen for the same task to be identical, these four models were finally picked as the fundamental learners. Their output vectors were merged to create a new feature matrix used as input for the second layer. Comprehensive explanations of these four fundamental models (GB, XGBoost, LightGBM, CatBoost) are available in the Supplementary Materials. Formula (27) represents the matrix output of the model:

(27) $$\mathrm{Output}\;\mathrm{Matrix}\;1=\left[Y_{1}{,Y}_{2},Y_{3},Y_{4}\right]$$

Various elementary learners employing combined nucleotide and amino acid coding techniques were utilized to provide prediction outcomes, resulting in a more extensive representation of sequence features. This approach effectively mitigates the correlation across models, hence enhancing the resilience and generalization capabilities of stacked models. Various models exhibit varying degrees of adaptation to different features of data, enabling them to capture a greater amount of information. The stacked model utilizes a feature selection method to automatically choose the most appropriate features from the model pool, allowing the model to determine which models are most effective for a specific task. This enhances the process of automating model selection and minimizes the requirement for manual involvement. Utilizing various fundamental learners enables the surpassing of constraints imposed by a solitary model and enhances the overall efficacy of the model. Each learner possesses distinct strengths and weaknesses, and by amalgamating them, the dispersion and divergence of each learner can be minimized, thereby improving generalization. Stacked models aggregate the predictions of individual learners in the second layer, thus mitigating the potential for overfitting that may occur with a single model. Various models within the model pool may exhibit overfitting tendencies in different respects, but their impacts might counterbalance one another during stacking, thereby mitigating the danger of overfitting. Ultimately, the utilization of this stacking technique for automatic selection enhances the efficiency and resilience of the model, streamlines the process of model selection, and amplifies the comprehensibility of the model.

The second layer of LncSL employed a meta-model, specifically logistic regression, to integrate the predictions obtained from the previous layer. The meta-model combines the results of each fundamental learner to acquire a more extensive and inclusive set of information. This approach maximizes the benefits and attributes of novice learners by merging their forecasts to yield more precise and dependable ultimate predictions. Logistic regression was selected as the meta-model due to its notable benefits. Logistic regression is straightforward and robust, easily implemented, and exhibits excellent computational efficiency, making it particularly valuable for handling large datasets. Additionally, it excels in handling binary output, making it especially suitable for classification tasks. Furthermore, logistic regression produces a probability score as its output, facilitating the comprehension and explanation of the model’s decision-making process. This score also enables the optimization of categorization outcomes by adjusting the threshold. Moreover, logistic regression typically employs maximum likelihood estimation to estimate its parameters, allowing the model to acquire and incorporate crucial features from the training data, thereby enhancing prediction accuracy. Simultaneously, the regularization capability of logistic regression enhances its robustness and effectively mitigates overfitting, a crucial aspect in integrated learning that preserves the diversity of fundamental learners. Thus, logistic regression was employed as a meta-model to enhance the accuracy and generalizability of predictions, while also boosting the interpretability and flexibility of the model. This robust integrated learning algorithm enhances the comprehensibility of the model and elucidates the decision-making process by automatically choosing the approach and employing the fundamental model and meta-model concurrently. The additional section contains a comprehensive explanation of the logistic regression meta-model employed in the second layer of LncSL. The meta-model’s prediction $\hat{y}$ is given by Formula (28):

(28) $$\hat{y}=\sigma \left(\sum_{k=1}^{m}\beta_{k}h_{k}\left(Z\right)+\beta_{0}\right)$$

where σ is the logistic sigmoid function, *β_k_* are the coefficients learned by the logistic regression model, and *m* is the number of basic learners.

LncSL has several advantages, including its extensive and varied feature coding methods, as well as its efficient automation and optimization in selecting basic learners and applying meta-models. To capture the extensive information in the lncRNA sequence, various feature coding approaches are employed, such as analyzing the properties of nucleotide and amino acid sequences. By incorporating multidimensional variables, the model’s prediction accuracy is improved, and its generalization capacity is enhanced. This surpasses the limitations of existing approaches that solely depend on the lncRNA sequence. Furthermore, originality is demonstrated in the choice of fundamental learners. LncSL utilizes a model pool consisting of numerous machine learning models to automatically identify the optimal features and select the most appropriate model for various tasks. This approach minimizes the requirement for manual intervention and greatly enhances the accuracy of predictions. Additionally, the likelihood of overfitting is mitigated by leveraging the benefits of numerous models. Ultimately, logistic regression is employed as a meta-model to further optimize the performance of the model. Logistic regression is an excellent option for meta-models due to its simplicity, high efficiency, and strong binary classification performance. Simultaneously, logistic regression produces a probability score as its output, which improves the comprehensibility of the decision-making process of the model and allows for flexibility in optimizing the classification results. Overall, this method offers a robust and extensively automated approach to stacking integrated learning. It achieves this through its novel feature coding, model selection, and meta-model application. This approach enhances the precision and resilience of the forecast and improves the comprehensibility of the model, offering a potent novel strategy for forecasting the subcellular localization of lncRNA. The process of model is shown in Algorithm 1.
**Algorithm 1:** Stacked ensemble learning for LncSL predictor**Input**: LncRNA sequence data**1. First Layer—Feature Encoding and Selection:**$F_{i}=FeatureEncodingMethod_{i}(sequenceData),\forall i$$FeatureMatrix=Combine(F_{1},F_{2},\ldots ,F_{n})$$FeatureSelectionMethods=\{Method_{1},Method_{2},\ldots ,Method_{7}\}$$FeatureSelectionResults_{j}=Method_{j}(FeatureMatrix),\forall j$$SelectedFeatures=Vote(FeatureSelectionResults_{1},\ldots ,FeatureSelectionResults_{7})$**2. First Layer—Basic Learner Training:**$M_{i}=TrainModel_{i}(SelectedFeatures),\forall i$$O_{i}=M_{i}(SelectedFeatures),\forall i$$ModelOutputMatrix=Combine(O_{1},O_{2},\ldots ,O_{m})$SelectedModels=BorutaModelSelection(ModelOutputMatrix)**3. Second Layer—Meta-Model Training:**MetaFeatureMatrix=Combine(SelectedModels)MetaModel=Train(LogisticRegression,MetaFeatureMatrix)FinalPrediction=MetaModel(TestDataModelOutputs)**Output**: *FinalPrediction*

#### 4.2.3. Evaluation Indicators

To comprehensively evaluate the performance of the model in this study, we used many evaluation indicators, including the Matthew’s correlation coefficient (MCC), receiver operating characteristic (ROC) curve, and confusion matrix. TP (true positive) represents accurate cytoplasm predictions, FP (false positive) represents incorrect cytoplasm predictions, TN (true negative) represents accurate nucleus predictions, and FN (false negative) represents incorrect nucleus predictions. Accuracy (ACC) is intuitive but can be misleading with unbalanced data. Sensitivity (SN) evaluates the model’s ability to detect true positives, while specificity (SP) assesses its ability to recognize true negatives. These indicators are interdependent. Average precision (AP) represents the average area under the precision–recall curve and is robust against class imbalance but less accurate with scarce positive samples. F1-scores emphasize classifier performance on minority classes in unbalanced dataset. The MCC is robust with unbalanced data, considering TP, TN, FP, and FN. Comprehensive indicators like ACC, AUC, MCC, and F1-scores provide an overall assessment of model performance. AUC correlates with the ROC curve, showing model performance under different thresholds, while MCC is particularly suitable for unbalanced datasets. Using multiple performance indicators allows for a thorough evaluation of model performance in predicting the subcellular localization of lncRNA. The calculation formulas for these evaluation indicators are shown in Formulas (29)–(36).

(29) $$\mathrm{Recall}=\frac{TP}{TP+FN}$$

(30) $$Precision=\frac{TP}{TP+FP}$$

(31) $$SP=\frac{TN}{TN+FP}$$

(32) $$AP=\int_{0}^{1}P\left(r\right)dr$$

(33) $$F1-\mathrm{score}=\frac{2\times TP}{2\times TP+FP+FN}$$

(34) $$ACC=\frac{TP+TN}{TP+FP+TN+FN}$$

(35) $$AUC=\int_{0}^{1}TPR\left(FPR\right)dFPR$$

(36) $$MCC=\frac{TP\times TN-FP\times FN}{\sqrt{\left(TP+FP\right)\left(TP+FN\right)\left(TN+FP\right)\left(TN+FN\right)}}$$

## 5. Conclusions

The function of long non-coding RNA (lncRNA) is heavily influenced by its precise cellular localization, which in turn impacts its involvement in regulating the cell cycle and rearranging the genome. Localization is a crucial requirement for comprehending the biological function and mechanism of lncRNA. Hence, accurately predicting the subcellular localization of lncRNA based on its sequence information has become a crucial step in uncovering its functional significance. This holds substantial scientific significance for further investigating the mechanism of action of lncRNAs in cells. The work aimed to create a novel method, namely LncSL, which can effectively forecast the subcellular positioning of lncRNA. LncSL differs from current approaches by using both the amino acid sequence features of open reading frame (ORF) translation and the lncRNA sequence itself. It utilizes a stacking model that is automatically chosen for training.

Furthermore, a diversified feature selection technique was employed, which involved the combination of seven distinct feature selection algorithms. This approach aimed to identify the most representative features and enhance the performance of the model. LncSL has demonstrated a 1.9% improvement in average precision (AP) compared to the existing predictive factors of TACOS, LncLocator2, GraphLncLoc, DeepLncLoc, and Locate-R, resulting in an AP of 24.4%. Additional evaluation metrics, including accuracy, F1-score, recall, Matthew’s correlation coefficient, and area under the curve, have demonstrated superior performance compared to current technology. These accomplishments confirm the efficacy of the LncSL algorithm and illustrate the significance of integrating sequence information with encoding potential features.

This study has offered a reliable method for properly predicting the subcellular localization of lncRNA. Bioinformatics has benefitted from the addition of novel research methodologies and viewpoints. The successful implementation of the LncSL algorithm showcases the capacity of computational biology to scrutinize intricate biological molecular pathways. This resource offers significant technical assistance and novel concepts for future investigations into the role of lncRNA and other interconnected biological matters. It is anticipated that in the future, this approach will be broadened to encompass a broader spectrum of lncRNA functional analysis and disease-related research, thereby enhancing our comprehension of the role of lncRNA in biology.

## Figures and Tables

**Table 1:** Prediction results of different feature codes on datasets.

Datasets	Feature	Recall	Precision	SP	ACC	F1-Score	MCC	AUC	AP
S1	CumulativeSkew	0.216	0.546	**0.846**	0.556	0.310	0.081	0.551	0.512
	PseDnc	0.479	0.571	0.692	0.594	0.521	0.175	0.634	0.584
	CTD	0.509	0.591	0.698	0.611	0.547	0.211	0.651	0.605
	Mer4	0.515	0.601	0.707	0.618	0.555	0.226	0.664	0.610
	PseKnc	0.501	0.587	0.699	0.607	0.541	0.203	0.645	0.590
	PseTranc	0.504	0.582	0.690	0.604	0.540	0.197	0.648	0.590
	PsePentanc	0.507	0.587	0.695	0.608	0.544	0.206	0.642	0.588
	ALL	**0.546**	**0.619**	0.712	**0.635**	**0.580**	**0.262**	**0.683**	**0.641**
S2	CumulativeSkew	0.271	0.581	**0.808**	0.542	0.370	0.094	0.560	0.571
	PseDnc	0.696	0.639	0.614	0.655	0.667	0.311	0.727	0.696
	CTD	0.676	0.659	0.656	0.666	0.667	0.332	0.732	0.708
	Mer4	0.735	0.685	0.667	0.701	0.709	0.403	0.767	0.748
	PseKnc	0.717	0.634	0.592	0.654	0.673	0.312	0.744	0.686
	PseTranc	0.678	0.668	0.669	0.674	0.673	0.348	0.744	0.707
	PsePentanc	0.628	0.648	0.665	0.646	0.638	0.293	0.711	0.671
	ALL	**0.737**	**0.690**	0.641	**0.705**	**0.713**	**0.412**	**0.770**	**0.765**

**Table 2:** Prediction results of the fusion of different amino acid feature codes on the datasets.

Datasets	Feature	Recall	Precision	SP	ACC	F1-Score	MCC	AUC	AP
S1	LncRNA + AAC	0.541	0.623	0.717	0.637	0.579	0.265	0.685	0.631
	LncRNA + CKS	0.556	0.617	0.705	0.636	0.585	0.264	0.688	0.628
	LncRNA + CKSAAP	0.548	0.614	0.705	0.633	0.579	0.256	0.692	0.644
	LncRNA + CTDT	0.536	0.612	0.708	0.629	0.572	0.249	0.684	0.625
	LncRNA + GAAC	0.548	0.623	0.716	0.638	0.583	0.266	0.680	0.623
	LncRNA + Geary	0.542	0.612	0.707	0.631	0.575	0.253	0.678	0.623
	LncRNA + SOCNumber	0.541	0.623	0.716	0.637	0.579	0.265	0.686	0.643
	LncRNA + TPC	0.559	0.625	0.713	0.642	0.590	0.267	0.696	0.640
	LncRNA + ZSCALE	0.542	0.622	0.718	0.637	0.579	0.264	0.689	0.637
	ALL-Extended	**0.555**	**0.639**	**0.732**	**0.650**	**0.594**	**0.276**	**0.698**	**0.654**
S2	LncRNA + AAC	0.727	0.684	0.671	0.698	0.705	0.398	0.779	0.761
	LncRNA + CKS	0.728	0.685	0.671	0.699	0.706	0.399	0.776	0.760
	LncRNA + CKSAAP	0.727	0.681	0.666	0.696	0.703	0.393	0.772	0.762
	LncRNA + CTDT	0.741	0.690	0.673	0.707	0.715	0.415	0.781	0.767
	LncRNA + GAAC	0.728	0.687	0.674	0.701	0.707	0.402	0.778	0.763
	LncRNA + Geary	0.735	0.691	0.677	0.706	0.712	0.412	0.781	0.756
	LncRNA + SOCNumber	0.729	0.681	0.664	0.696	0.704	0.393	0.778	0.763
	LncRNA + TPC	0.736	0.694	0.680	0.708	0.714	0.417	0.777	0.757
	LncRNA + ZSCALE	0.733	0.687	0.672	0.702	0.709	0.405	0.779	0.770
	ALL-Extended	**0.735**	**0.708**	**0.701**	**0.718**	**0.721**	**0.437**	0.776	**0.770**

**Table 3:** Comparison between two datasets before and after feature selection.

Datasets	Feature Selection	Recall	Precision	SP	ACC	F1-Score	MCC	AUC	AP	Time
S1	Yes	0.559	0.628	0.717	0.649	0.591	**0.279**	0.682	0.632	**7.0 m**
	No	0.555	0.639	0.732	0.650	0.594	0.276	0.698	0.654	154.1 m
S2	Yes	0.723	0.709	0.714	0.719	0.718	**0.437**	0.778	0.767	**2.3 m**
	No	0.735	0.708	0.701	0.718	0.721	0.437	0.776	0.756	344.3 m

**Table 4:** Comparison between LncSL and single model.

Datasets	Feature	Recall	Precision	SP	ACC	F1-Score	MCC	AUC	AP
S1	GB	0.535	0.607	0.703	0.626	0.569	0.242	0.670	0.625
	LGBM	0.542	0.600	0.690	0.622	0.570	0.235	0.660	0.612
	XGB	0.544	0.601	0.691	0.623	0.571	0.238	0.667	0.626
	CatBoost	**0.559**	0.628	0.717	0.649	0.591	0.279	0.682	0.632
	LncSL	0.556	**0.639**	**0.732**	**0.650**	**0.594**	**0.292**	**0.702**	**0.666**
S2	GB	0.748	0.683	0.658	0.703	0.715	0.409	0.775	0.756
	LGBM	0.716	0.711	**0.713**	0.714	0.713	0.429	0.775	0.752
	XGB	0.717	0.708	0.710	0.713	0.713	0.427	0.775	0.756
	CatBoost	0.723	0.709	0.714	0.719	0.719	0.437	0.778	0.767
	LncSL	**0.749**	**0.710**	0.700	**0.724**	**0.729**	**0.449**	**0.794**	**0.782**

**Table 5:** Comparison of LncSL with other predictive factors.

Datasets	Methods	Recall	Precision	SP	ACC	F1-Score	MCC	AUC	AP
S1	TACOS	0.516	0.559	0.651	0.589	0.536	0.168	0.606	0.540
	LncLocator2	0.210	**0.666**	**0.910**	0.587	0.319	0.169	0.642	0.597
	GraphLncLoc	0.427	0.494	0.626	0.534	0.458	0.054	0.539	0.485
	DeepLncLoc	0.489	0.565	0.678	0.591	0.524	0.169	0.626	0.546
	Locate-R	0.152	0.556	0.896	0.553	0.239	0.072	0.554	0.514
	LncSL	**0.556**	0.639	0.732	**0.650**	**0.594**	**0.292**	**0.702**	**0.666**
S2	TACOS	0.653	0.659	0.668	0.661	0.656	0.321	0.719	0.687
	LncLocator2	**0.817**	0.643	0.555	0.685	0.720	0.385	0.727	0.695
	GraphLncLoc	0.402	0.548	0.674	0.539	0.464	0.080	0.566	0.538
	DeepLncLoc	0.724	0.678	0.661	0.692	0.700	0.386	0.757	0.763
	Locate-R	0.540	0.629	0.687	0.614	0.581	0.230	0.651	0.653
	LncSL	0.749	**0.710**	**0.700**	**0.724**	**0.729**	**0.449**	**0.794**	**0.782**

**Table 6:** Results of different predictive factors on independent test dataset.

Methods	Recall	Precision	SP	ACC	F1-Score	MCC	AUC	AP
TACOS	0.520	0.140	0.740	0.724	0.221	0.153	0.668	0.115
LncLocator2	0.163	**0.180**	**0.940**	**0.881**	0.171	0.108	0.649	0.133
GraphLncLoc	0.264	0.081	0.759	0.721	0.124	0.014	0.505	0.078
DeepLncLoc	**0.594**	0.106	0.594	0.594	0.180	0.100	0.619	0.101
Locate-R	0.161	0.109	0.893	0.838	0.130	0.046	0.570	0.094
LncSL	0.524	0.169	0.790	0.770	**0.258**	**0.200**	**0.774**	**0.140**

**Table 7:** Nuclear and cytoplasmic distribution of all datasets.

Datasets	Cytoplasm	Nucleus	Total
S1	10,351	12,088	22,439
S2	4150	4218	8368
independent	1127	13,846	14,991

## Data Availability

Publicly available datasets were analyzed in this study. Codes and data are available at https://github.com/ChenHongCCZU/LncSL (accessed on 20 December 2024).

## Supplementary Materials

The following supporting information can be downloaded at: https://www.mdpi.com/article/10.3390/ijms252413734/s1. References [52,53,54,55,56] are cited in the Supplementary Materials.
