# Supplementary material for: LncSL: A Novel Stacked Ensemble Computing Tool for Subcellular Localization of lncRNA by Amino Acid-Enhanced Features and Two-Stage Automated Selection Strategy
Source: Int J Mol Sci. 2024 Dec 23;25(24):13734. doi: 10.3390/ijms252413734 (PMC11678684; doi:10.3390/ijms252413734)
Supplement: Supplementary file 1 [file ijms-25-13734-s001.zip › ijms-3326498-supplementary.pdf]

Supplementary

1. Random Forest (RF)

In 2016, Belgiu *et al*[1]. utilized random forests (RF) for classification and regression. RF combines predictions from multiple independent decision trees, aiming to enhance model generalization. It constructs diverse trees by randomly selecting data samples and subsets of features. This strategy reduces variance and improves performance on new data. The mathematical expression of RF is shown in formula 1:

$$F\left(X\right) = arg\max_{y}\sum_{i=1}^{k} I\left(f_i\left(X\right) = y\right) \tag{1}$$

Among them, $F\left(X\right)$ represents the composite classification model, I represents the indicator function, f_i represents the decision tree classification model, and y represents the outcome variable. In this study, RF was used as an estimator for Boruta feature selection due to its adaptability in handling high-dimensional data and complex relationships. RF was applied to our nucleotide binding amino acid feature space, which has a large dimension of 12099, making it an effective key feature selection method for our LncRNA subcellular localization task.

2. Logistic Regression (LR)

Logistic Regression (LR) is a statistical learning method for classification[2]. It's a type of Generalized Linear Model (GLM), primarily for binary classification but extendable to multi-class tasks. In this study, LR served as a meta-classifier for stacking learning to integrate base classifier predictions. LR's basic form converts linear regression output into probabilities via a logistic function, representing class probabilities. Formula 2 can represent this:

$$h\left(x\right) = b + w_1 x_1 + \ldots + w_n x_n \tag{2}$$

Among them, x_i is the input feature with weight parameters, and b is the bias value. Given an unmarked input x , the possibility of x being associated with a class label (given PTM type) can be defined as formula 3:

$$P\left(h\left(x\right)\right) = \frac{1}{1 + e^{-h(x)}} \tag{3}$$

In this study, logistic regression (LR) was used as the meta-classifier for stacked learning to combine base classifier predictions. Preliminary predictions for lncRNA subcellular localization from other ML algorithms served as input features for LR. With four first-level basic learners, the feature dimension is 4, making LR's simplicity advantageous by reducing overfitting risks. LR optimizes parameters by training on the first layer output, ultimately providing the final subcellular localization predictions.

3. Gradient Boosting Machine(GBM)

GBM is a flexible ensemble learning technique, that combines gradient-based optimization and boosting methods. Gradient-based optimization minimizes the model's loss function concerning training data by computing gradients while boosting iteratively assembles "weak learners" to form a robust prediction model, ideal for classification tasks[3]. This algorithm can automatically select features, prioritize important variables, and discard irrelevant or redundant ones. Represented as a linear combination of models, GBM is expressed in formula 4:

$$y = 1\mu + \vartheta_1 h_1(y;X) + \vartheta_2 h_2(y;X) + \vartheta_3 h_3(y;X) + \vartheta_m h_m(y;X) + \ldots + \vartheta_M h_M(y;X) + e \quad (4)$$

Among them, y is the observation vector, μ is the intercept, $h_m(y;X)$ ($m \in \{1,\ldots,M\}$) represents each model applied, ϑ_m ($m \in \{1,\ldots,M\}$) is the weight parameter applied to each model, and e is the residual vector. Initially, the algorithm fits the first model to the residual of the initialization prediction based on the response variable distribution. Subsequently, the algorithm fits each model's residual to the next model. At this stage, the residual of model m can be viewed as the residual estimate $(\hat{e})$, where $\hat{e} \sim N(0, \sigma_{e_m}^2)$, $\sigma_{e_m}^2$ are the residual variances of model m. Initially, the algorithm fits the first model to the residuals of the initial prediction. Each subsequent model fits its residuals to the next model, minimizing prediction error until no further improvement is possible, resulting in model M. We integrated traditional nucleotide sequence features and derived amino acid enhancement features. GBM effectively handles diverse data features and uncovers nonlinear relationships, which is crucial for our lncRNA subcellular localization task.

4. eXtreme Gradient Boosting(XGB)

XGBoost (eXtreme Gradient Boosting)[4] is an advanced ensemble learning method that builds on GBDT (Gradient Boosting Decision Tree) technology. It improves model accuracy and robustness by incorporating regularization components and second-order derivative information. XGBoost uses weighted decision trees and fine-tunes the objective function for optimal performance. Its goal is to build k regression trees for accurate prediction and strong generalization, as illustrated by its objective function, as shown in formula 5:

$$L(\phi) = \sum_i l(\bar{y} - y_i) + \sum_k o(f_k) \quad (5)$$

Where i represents the i-th sample, $l(\bar{y} - y_i)$ represents the predicted difference of the i-th sample, and $\sum_k o(f_k)$ represents the complexity function of the tree.

In the XGBoost algorithm, optimizing the objective function is key. This function includes the loss function, which measures prediction error, and the regularization term, which prevents overfitting by limiting model complexity. The objective function sums the loss functions of all decision trees, adding regularization penalties for each tree's complexity. XGBoost minimizes this function through gradient boosting, sequentially stacking tree models. It adjusts the loss function and regularization for specific bioinformatics problems. In our lncRNA subcellular localization prediction, XGBoost handles noise and missing sequence information (denoted by N) by filtering out these values and automatically managing missing data, ensuring robustness and accuracy.

5.LigthGBM

LightGBM[5] is a Boosting-based ensemble learning algorithm, derived from GBDT, known for reducing training time and memory usage without sacrificing accuracy. It uses Gradient Single Side Sampling (GOSS) to prioritize data with larger gradients and Exclusive Feature Bundling (EFB) to reduce dimensionality. LightGBM also employs a histogram algorithm and depth-limited leaf growth strategy to minimize memory use. The histogram algorithm discretizes continuous features into histograms for efficient data traversal and optimal segmentation. The depth-limited leaf growth strategy selects the leaf with the highest splitting gain, imposing a maximum depth to prevent overfitting. Like XGBoost, LightGBM's objective function includes a loss function and a regularization term (L1+L2), utilizing the second-order Taylor expansion for approximation, as depicted in formulas 6 and 7:

$$Obj^t \approx \sum_{i=1}^{N}\left[l\left(y_i,\hat{y}_i^{t-1}\right)+g_i f_t\left(x_i\right)+\frac{1}{2}h_i f_t^2\left(x_i\right)\right]+\Omega\left(f_t\right) \tag{6}$$

$$Among\, them g_i = \partial_{\hat{y}_i^{t-1}} l\left(y_i,\hat{y}_i^{t-1}\right), h_i = \partial_{\hat{y}_i^{t-1}}^2 l\left(y_i,\hat{y}_i^{t-1}\right) \tag{7}$$

The subcellular localization task for lncRNA involves numerous features and samples, making LightGBM ideal due to its efficiency in memory usage and model training. Its histogram decision tree algorithm and GOSS (Gradient Single Side Sampling) enhance performance by focusing on high-gradient data, which is crucial for identifying important features in the presence of many irrelevant ones. EFB (Exclusive Feature Bundling) reduces dimensionality and time complexity, streamlining the model without compromising accuracy. LightGBM's gradient boosting and leaf-first tree growth approach is adept at handling intricate features like CumulateSkew and CTD, capturing complex patterns and interactions, thus improving prediction accuracy and efficiency for lncRNA subcellular localization tasks.

6. Categorical Boosting

CatBoost, introduced by Hancock *et al*[6]. in 2020, is a gradient-boosted decision tree technique using symmetric trees and accommodating categorical variables. It has fewer parameters than other algorithms, enhancing accuracy. Traditional GBDT algorithms often suffer from conditional biases with classification features. CatBoost addresses this by enhancing the greedy TS method with a prior term and weighting factor, as shown in Equation 8:

$$x_k^i = \frac{\sum_{j=1}^{p-1}\left[x_{\sigma_{j,k}} = x_{\sigma_{p,k}}\right] Y\sigma_j + \alpha \times p}{\sum_{j=1}^{p-1}\left[x_{\sigma_{j,k}} = x_{\sigma_{p,k}}\right] + \alpha} \tag{8}$$

CatBoost excels in handling categorical features by automatically converting them to numerical form, eliminating the need for manual encoding. This is advantageous for biological data like gene expression patterns and RNA sequences, which are rich in categorical information. By modifying the greedy TS method and introducing prior terms and weighting factors, CatBoost addresses conditional deviations better than traditional GBDT, crucial for lncRNA subcellular localization tasks with complex biological processes. Its specialized design mitigates overfitting, ensuring model generalization. CatBoost is also effective in handling high-dimensional, sparse feature spaces with many zeros, like K-mer features. Its optimized structure processes numerous features efficiently, capturing complex interdependencies and improving prediction accuracy. Additionally, CatBoost mitigates the impact of noise and incomplete information in lncRNA data, enhancing the discovery

of vital biological patterns.

References

1. Belgiu M, Drăguţ L (2016) Random forest in remote sensing: A review of applications and future directions. ISPRS Journal of Photogrammetry and Remote Sensing 114:24–31. https://doi.org/10.1016/j.isprsjprs.2016.01.011

2. LaValley MP (2008) Logistic Regression. Circulation 117:2395–2399. https://doi.org/10.1161/CIRCULATIONAHA.106.682658

3. Natekin A, Knoll A (2013) Gradient boosting machines, a tutorial. Front Neurorobot 7:. https://doi.org/10.3389/fnbot.2013.00021

4. Shin H (2022) XGBoost Regression of the Most Significant Photoplethysmogram Features for Assessing Vascular Aging. IEEE J Biomed Health Inform 26:3354–3361. https://doi.org/10.1109/JBHI.2022.3151091

5. Chen C, Zhang Q, Ma Q, Yu B (2019) LightGBM-PPI: Predicting protein-protein interactions through LightGBM with multi-information fusion. Chemometrics and Intelligent Laboratory Systems 191:54–64. https://doi.org/10.1016/j.chemolab.2019.06.003

6. Hancock JT, Khoshgoftaar TM (2020) CatBoost for big data: an interdisciplinary review. J Big Data 7:94. https://doi.org/10.1186/s40537-020-00369-8
